# Deficiency of a Niemann-Pick, Type C1-related Protein in *Toxoplasma* Is Associated with Multiple Lipidoses and Increased Pathogenicity

**DOI:** 10.1371/journal.ppat.1002410

**Published:** 2011-12-08

**Authors:** Bao Lige, Julia D. Romano, Veera Venkata Ratnam Bandaru, Karen Ehrenman, Jelena Levitskaya, Vera Sampels, Norman J. Haughey, Isabelle Coppens

**Affiliations:** 1 Department of Molecular Microbiology and Immunology, Johns Hopkins University Bloomberg School of Public Health, Baltimore, Maryland, United States of America; 2 Department of Neurology, Division of Neuroimmunology and Neurological Infections, Johns Hopkins University School of Medicine, Baltimore, Maryland, United States of America; University of Geneva, Switzerland

## Abstract

Several proteins that play key roles in cholesterol synthesis, regulation, trafficking and signaling are united by sharing the phylogenetically conserved ‘sterol-sensing domain’ (SSD). The intracellular parasite *Toxoplasma* possesses at least one gene coding for a protein containing the canonical SSD. We investigated the role of this protein to provide information on lipid regulatory mechanisms in the parasite. The protein sequence predicts an uncharacterized Niemann-Pick, type C1-related protein (NPC1) with significant identity to human NPC1, and it contains many residues implicated in human NPC disease. We named this NPC1-related protein, TgNCR1. Mammalian NPC1 localizes to endo-lysosomes and promotes the movement of sterols and sphingolipids across the membranes of these organelles. Miscoding patient mutations in NPC1 cause overloading of these lipids in endo-lysosomes. TgNCR1, however, lacks endosomal targeting signals, and localizes to flattened vesicles beneath the plasma membrane of *Toxoplasma*. When expressed in mammalian NPC1 mutant cells and properly addressed to endo-lysosomes, TgNCR1 restores cholesterol and GM1 clearance from these organelles. To clarify the role of TgNCR1 in the parasite, we genetically disrupted *NCR1*; mutant parasites were viable. Quantitative lipidomic analyses on the ΔNCR1 strain reveal normal cholesterol levels but an overaccumulation of several species of cholesteryl esters, sphingomyelins and ceramides. ΔNCR1 parasites are also characterized by abundant storage lipid bodies and long membranous tubules derived from their parasitophorous vacuoles. Interestingly, these mutants can generate multiple daughters per single mother cell at high frequencies, allowing fast replication in vitro, and they are slightly more virulent in mice than the parental strain. These data suggest that the ΔNCR1 strain has lost the ability to control the intracellular levels of several lipids, which subsequently results in the stimulation of lipid storage, membrane biosynthesis and parasite division. Based on these observations, we ascribe a role for TgNCR1 in lipid homeostasis in *Toxoplasma*.

## Author Summary

The intracellular parasite *Toxoplasma* is auxotrophic for several lipids that it scavenges from host organelles. Several studies focused on deciphering the pathways implicated in host lipid delivery to the parasite, but less effort has been devoted to understand how lipids are regulated in *Toxoplasma*. The ‘sterol-sensing domain’ (SSD) is conserved across phyla and present in several membrane proteins involved in cholesterol homeostasis, cell signaling and cytokinesis. We studied the role of a SSD-containing protein in *Toxoplasma* which shows significant similarity with Niemann-Pick type C1 proteins (NPC1). Human NPC disease is typified by lysosomal accumulation of cholesterol and sphingolipids. Expression of the parasite NPC1-related protein (named TgNCR1) in mammalian NPC1 mutant cells suppresses lipid accumulation in lysosomes. However, *Toxoplasma* never internalizes host cholesterol into lysosomes, which predicts a function for TgNCR1 unrelated to exogenous sterol transport. Indeed, genomic deletion of *NCR1* does not result in abnormal levels of cholesterol in the parasites but in the overaccumulation of cholesteryl esters and sphingolipids. TgNCR1-deficient parasites form abundant storage lipid bodies and multiple parasites per cycle of division. This suggests that TgNCR1 functions in monitoring the levels of various lipids within *Toxoplasma*, which in turn impacts the parasite's lipid homeostasis and growth rate.

## Introduction

*Toxoplasma gondii* is an obligate intracellular parasite that resides in a unique vacuole formed in the cytoplasm of mammalian cells during invasion. The parasitophorous vacuole (PV) of *Toxoplasma* protects the parasite from hostile cytosolic innate immune-surveillance pathways and potent inflammatory signaling cascades. Although separated from the nutrient-rich cytosol by the PV membrane, the parasite has nevertheless evolved efficient strategies to co-opt multiple host cellular pathways and host organelles, to acquire essential nutrients and fuel its growth. The parasite expresses many surface transporters that mediate the internalization of host molecules [1]. *T. gondii* contains large amounts of cholesterol that it scavenges from plasma low-density lipoproteins (LDL) after processing in host endocytic compartments [2]. Interference with LDL endocytosis or cholesterol egress from host lysosomes arrests parasite development. We demonstrated that *Toxoplasma* can sequester nutrient-filled host lysosomes within invaginations of the PV membrane, which allows access to cholesterol supplied by the host endocytic network [3]. Cholesterol incorporation into the parasite is abolished after treatment with various proteases [4], and we have recently identified a transport system of cholesterol to the parasite involving parasite ABCG proteins [5]. Although much is known about host cholesterol delivery to *Toxoplasma*, very little is known about the regulatory mechanisms and trafficking routes of cholesterol (and other lipids) within the parasite. We have reported a role for a D-bifunctional protein containing two sterol-carrier protein-2 domains in promoting the circulation of phospholipids, cholesterol and fatty acids between parasite organelles and the plasma membrane in *T. gondii* [6].

In higher organisms, intracellular cholesterol transport is a fundamental process required for cell division, growth and differentiation. The distribution of cholesterol in different subcellular compartments is maintained by a combination of vesicle-mediated interorganelle transport and protein-mediated monomeric transfer through the aqueous cytosol [7], [8]. Among the cholesterol-binding proteins, the Niemann-Pick C (NPC) proteins, NPC1 and NPC2, are required for cholesterol export from endocytic organelles [9]. Loss of function of either of these proteins causes the sequestration of LDL-derived cholesterol and other lipids in these organelles and leads to a progressive neurodegenerative disorder: the NPC disease [10]. NPC1 is a multispanning transmembrane protein residing predominantly in the limiting membrane of late endosomes/lysosomes while NPC2 is a soluble lysosomal protein [11], [12]. Both proteins operate in the trafficking of cholesterol in the endo-lysosomal vesicle system; cholesterol liberated from LDL is first bound to NPC2 that then hands off the lipid to NPC1 that expels cholesterol out of the lysosomal compartment [13], [14]. NPC2 has a hydrophobic interior containing small cavities that can accommodate the steroid core while NPC1 has a high affinity binding domain to cholesterol at the N-terminus, called the sterol-binding domain (SBD). In addition, NPC1 has five to six transmembrane regions constituting the putative ‘sterol-sensing domain’ (SSD) that is common to many proteins that have key roles in cholesterol homeostasis or cholesterol-linked signaling [15]. However, while several potential interaction surfaces of the NPC proteins have been identified, the molecular mechanism of NPC interaction to transfer cholesterol remains still elusive.

NPC1 and NPC2 are conserved throughout much of eukaryotic evolution [16]. An authentic NPC1 ortholog appears in fungi, worms, insects, slime molds, plants and all mammals. Within the genome of most multicellular organisms, the *NPC1* gene is represented at least twice. The retention of *NPC1* genes throughout eukaryotic evolution permits the identification of conserved sequence motifs that include the SSD [15]. In addition to NPC1, 3-hydroxy-3-methylglutaryl CoA reductase (HMGCoA reductase, the key enzyme of the mevalonate pathway producing sterols), SCAP [the sterol regulatory element-binding protein (SREBP)-cleavage activating protein] (a regulator of the sterol-dependent transcription of cholesterol biosynthetic genes), 7-dehydrocholesterol reductase (an enzyme involved in cholesterol biosynthesis), Patched (a tumor suppressor involved in the signal transduction cascade mediated by the cholesterol-modified morphogen Hedgehog), Dispatched (a protein that facilitates the secretion of Hedgehog) and the Patched-related protein (a protein closely related in sequence and predicted topology to Patched) share a SSD. Mutations in the SSD of these proteins in mammalian cells render insensitivity not only to sterols but also to other lipids such as oxysterols, fatty acids, sphingolipids and phospholipids, suggesting that the SSD is a motif for membrane anchorage that can sense and respond to various agents that perturb membranes [17]–[20]. In yeast, a dominant mutation in the putative SSD of NCR1, a homolog of NPC1, confers resistance to inhibitors of inositophosphorylceramide and accumulation of complex sphingolipids, but no defect in sterol metabolism, pointing to a primary role of yeast NCR1 in sphingolipid recycling instead of sterol homeostasis [21].

To gain more insight into the key cellular pathways that govern lipids, e.g., cholesterol movement within *T. gondii*, we have characterized a parasite protein harboring a canonical SSD. This protein is a close relative of human NPC1, and we named it TgNCR1. When targeted to mammalian endo-lysosomes, TgNCR1 restores lipid trafficking from these organelles in mammalian NPC1 mutant cells. We generated a parasite cell line lacking *NPC1* that is viable. The mutant parasites exhibit perturbations in their content in lipids, e.g. cholesteryl esters and sphingolipids, but not in free cholesterol. These alterations are reflected by the amassing of large lipid bodies in the parasite cytoplasm, and the stimulation of membrane biosynthesis and parasite replication. This suggests the involvement of TgNCR1 in monitoring the status of various lipids in *Toxoplasma*, a regulatory function likely important for proper parasite growth.

## Results

### Cloning and molecular characterization of a *Toxoplasma* protein that harbors an archetypal ‘sterol sensing-like domain’ and shares identity with human NPC1

To identify parasite proteins involved in cholesterol homeostasis in *T. gondii*, we searched for sequence homology to the sterol-sensing domain (SSD) in the *Toxoplasma* genome database (ToxoDB.org). A gene coding for a transmembrane polypeptide containing a sterol-sensing-like domain, annotated ‘Patched transmembrane domain-containing protein’ was retrieved (TGME49_090870; location on chromosome IX). Another gene with the same annotation was also present but its expression level was predicted to be very low (TGME49_120500; location on chromosome IV). As expected, no genes with homologous sequences to sterol biosynthetic enzymes, HMGCoA reductase and 7-dehydrocholesterol reductase, could be identified from the genomic database of *T. gondii*. The regulatory SREBP-SCAP machinery and the Patched/Hedgehog system are also missing from the parasite genome. The ORF of TGME49_090870 was amplified by PCR from a sporozoite cDNA library; it is 3,534 nucleotides long, consists of 6 exons and encodes a polypeptide of 1,178 amino acids, predicting a protein of 130.6-kDa (Figure S1). A parallel search of the Eukaryotic Pathogens Database Resources (EuPathDB.org) for sequence homology to TGME49_090870 using Jalview 2.6.1 [22] revealed the presence of one or two versions of NPC1-related protein in several apicomplexan parasites including *Neospora caninum*, *Cryptosporidium* sp. and *Plasmodium* sp. but no NPC1 homolog in any species of *Theileria* or *Babesia* (Figure S2).

**Figure 1:**
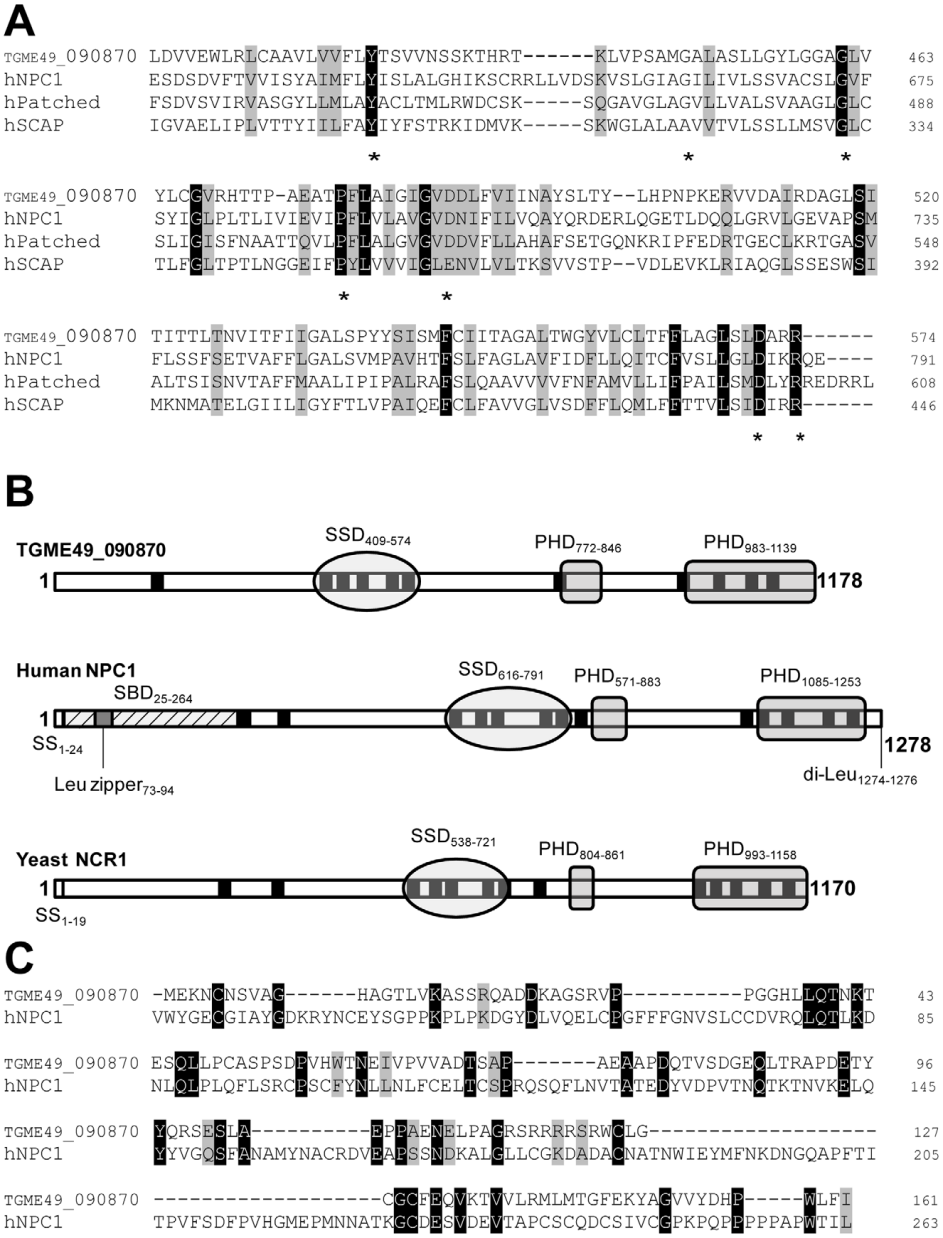
Characteristic features of the predicted sequence of TGME49_090870 displaying a sterol-sensing-like domain and some conserved critical motifs of the NPC1 family. A. Multiple sequence alignment of the predicted SSD of TGME49_090870 with the human sequences of NPC1 (GI:38649260), Patched (GI:1381236) and SCAP (GI:66932902) using the CLUSTALW program, revealing the presence of many conserved identical (black boxes) and similar (grey boxes) amino acids in these proteins. Asterisks show some amino acids that are critical for SSD function and responsible for the human NPC disease if mutated or missing. B. Schematic of the domain structure of TGME49_090870 (obtained after cloning), human NPC1 and yeast Ncr1p (GI:259150148), showing the position of the predicted SSD, PHD (Patched Homology Domain) and transmembrane domains (in black). The sequence of TGME49_090870 lacks the Leu zipper present in the high affinity sterol-binding domain (SBD) and the endosomal targeting motifs identified in the human NPC1 sequence. C. Sequence alignment of the predicted N-terminal sequences of TGME49_090870 and human NPC1 comprising the SBD, showing a high proportion of identical amino acids.

Analysis of the deduced amino acid sequence of the sterol sensing-like domain of TGME49_090870 reveals high similarity to the SSD of the members of the cholesterol-sensing protein family (Figure 1A). The parasite sterol sensing-like domain encompasses 180 amino acids and is organized into five membrane spanning α-helices (Figure S3). This domain shares 31% identity and 51% similarity with the SSD of human NPC1. Interestingly, the sterol sensing-like domain in the *Toxoplasma* protein contains many residues implicated in the human NPC disease [23]–[26]. Of the miscoding mutations identified in the SSD of human NPC1 that are responsible for severe biochemical defects in patients, seven amino acids (70%) are identical between the parasite protein and human NPC1 (Y-634, G-660, G-673, P-691, D-700, D-786 and R-789). Figure 1A highlights the SSD containing a number of conserved amino acids that have been implicated in the NPC disease and that are present in the *Toxoplasma* protein.

**Figure 2:**
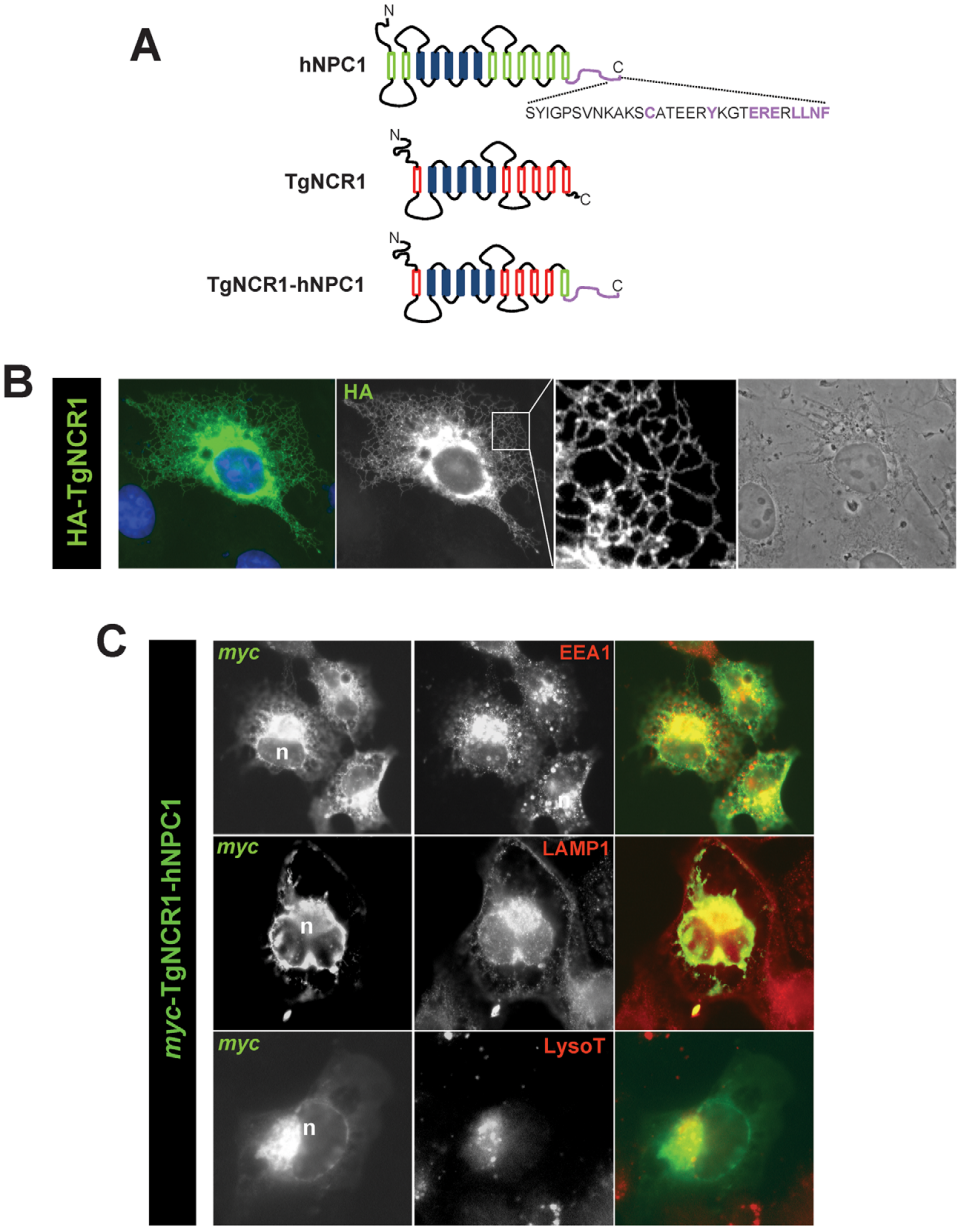
Localization of TgNCR1 in mammalian cells after transfection. A. Schematic representation of the NPC1 constructs: human NPC1 (hNPC1) showing the endosomal targeting motif in purple, full lengthTgNCR1 or TgNCR1 modified in which the last 78 amino acids have been deleted and replaced by the last 64 amino acids of hNPC1, designed TgNCRC1-hNPC1. B–C. Fluorescence microscopy of CHO cells expressing the TgNCR1 constructs with either HA or *myc* tags at the N-terminus. After transfection of p*HA-TgNCR1* in B or p*myc-TgNCR1-hNPC1* in C, cells were fixed and immunostained with anti-HA or anti-*myc* antibodies to localize the TgNCR1 constructs. A pattern corresponding to the ER was identified for TgNCR1 while TgNCR1-hNPC1 was mainly expressed in endo-lysosomal compartments, which were identified by immunostaining of endogenous markers (EEA1, an early endosome-associated protein or LAMP1, a lysosomal-associated membrane protein), or by labeling with Lyso-Tracker (LysoT). In blue, DAPI staining; n, nucleus.

In addition to a putative SSD, TGME49_090870 has multiple membrane spanning domains and two Patched Homology Domains (PHD) that typify the NPC family (Figure 1B). The parasite sequence does not possess endosomal targeting signals such as a C-terminal di-Leu motif [27], probably due to the lack of endocytic compartments in the parasite. The N-terminal sequence of human NPC1 harbors the sterol-binding domain (SBD) on luminal loop-1 that is known to interact with cholesterol and oxysterols with high affinity [28], [29]. Sequence alignment of the N-terminal ends of TGME49_090870 and human NPC1 encompassing the SBD reveals the presence of 35 identical residues (22%), which suggests that the N-terminus of the parasite protein may have potential sterol binding activity (Figure 1C). Moreover, several conserved Pro and Gly residues present in the parasite amino-terminal sequence are critical residues whose substitutions correspond to naturally occurring mutations in patients with NPC disease. At the protein level, the parasite full-length sequence has 37% identity and 61% similarity with human NPC1; 61 of the functionally important residues (59%) for the sterol transfer function are present, and 47 of those are identical to human NPC1. Based on these observations, we therefore renamed the protein, TgNCR1 (for *N*P*C*-*r*elated gene *1*). A schematic representation of the topology of TgNCR1 and human NPC1 shown in Figure 2A highlights their structural similarities.

### Expression of TgNCR1 corrects the cholesterol-trafficking defect in NPC1 mutant cells

To test the functional equivalence of TgNCR1 and mammalian NPC1, we planned to express TgNCR1 in a CHO cell line lacking functional *NPC1* and examine the potential ability of the parasite protein to restore NPC1 activity in the mutant cells, namely the abatement of lipid accumulation in endo-lysosomes. However, as *Toxoplasma* does not possess an endocytic membranous system involved in the internalization of extracellular cholesterol, TgNCR1 does not contain any targeting information for endocytic organelles as found on human NPC1. We first analyzed the intracellular localization of TgNCR1 when ectopically expressed in mammalian cells. CHO cells were transfected with pHA-TgNCR1 and immunolabeled with antibodies against HA (Figure 2B). A fluorescence pattern corresponding to cortical and perinuclear ER was discernible. Expectedly, no staining was observed on endo-lysosomal compartments. We therefore decided to engineer a chimeric protein to correctly address TgNCR1 to endo-lysosomes in mammalian cells. Mammalian NPC1 localization to endo-lysosomes is mediated by several COOH-terminal motifs [26]. We constructed a hybrid protein in which the last 64 residues of human NPC1 that encompass most of the functional endosomal targeting motifs (hNPC1_1214-1278_) were fused to TgNCR1 lacking its last 78 amino acids (TgNCR1_Δ1101-1178_) as schematized in Figure 2A. A *myc* tag was introduced at the N-terminus of the chimeric protein designated *myc*-TgNCR1-hNPC1. The expression construct was transfected into CHO cells for double immunofluorescence assays (IFA) using antibodies against *myc* and various markers of endosomes or lysosomes (Figure 2C). Results show that the TgNCR1-hNPC1 fusion protein largely localized to structures positively labeled for EEA1, LAMP1 or LysoTracker, indicating delivery of the exogenous protein to endocytic organelles.

**Figure 3:**
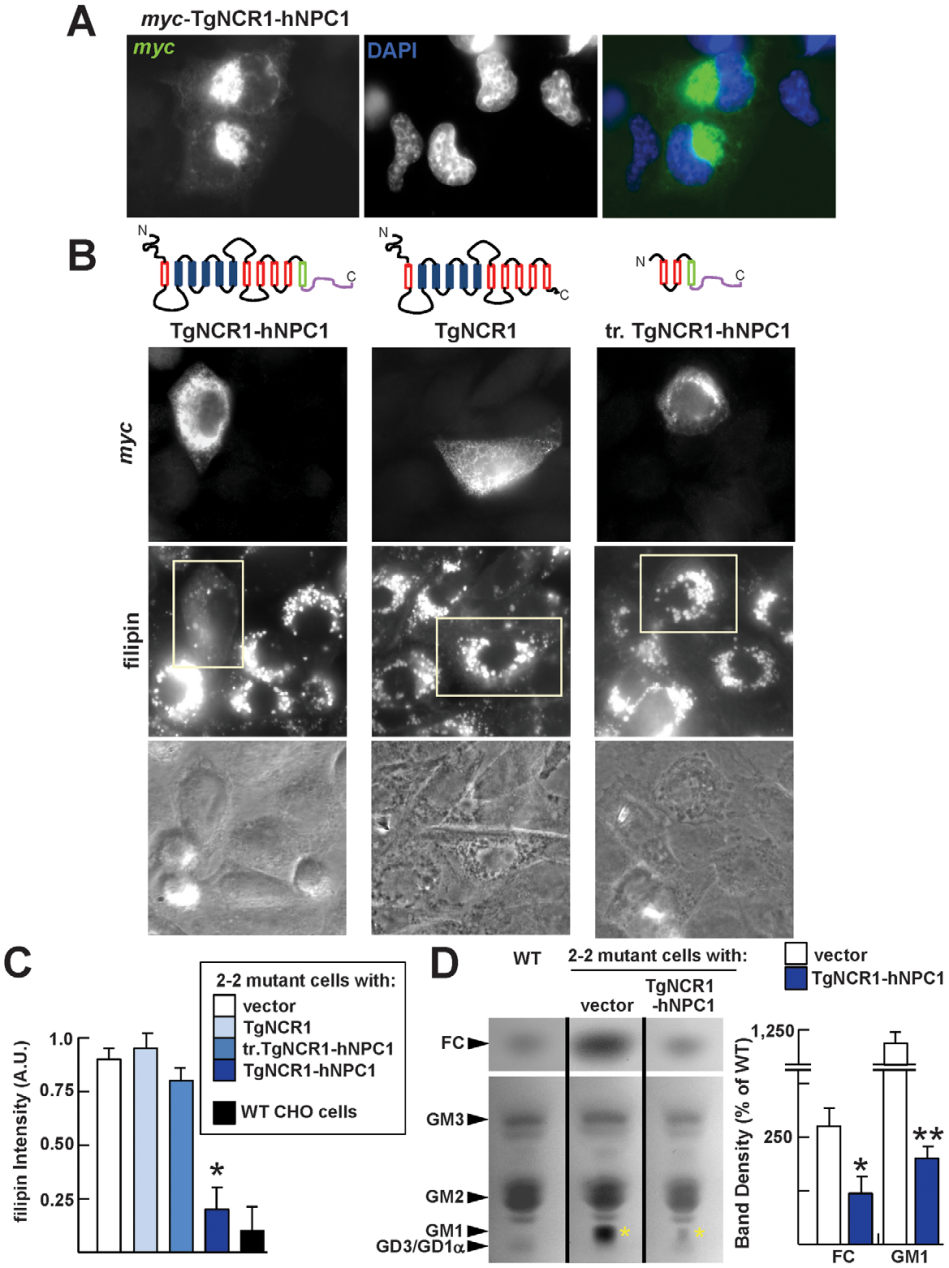
Decrease in sterol accumulation in NPC1 mutant cells expressing TgNCR1-hNPC1. A. Immunofluorescence microscopy of 2-2 mutant CHO cells expressing TgNCR1-hNPC1. 2-2 mutant cells have been transiently transfected with p*myc-TgNCR1-hNPC1*, fixed and immunostained using anti-*myc* antibodies. *Myc*-TgNCR1-hNPC1 shows staining in perinuclear lysosomes. B. Filipin staining of 2-2 mutant cells expressing TgNCR1-hNPC1, TgNCR1 full-length or a truncated version of the C-terminal end of TgNCR1-hNPC1 (tr. TgNCR1-hNPC1). After transient transfection with p*myc-TgNCR1-hNPC1*, p*myc*-TgNCR1 or p*myc-tr.TgNCR1-hNPC1*, cells were stained with anti-myc antibodies to detect tagged protein expression and with filipin to visualize sterols in transfected cells. Representative pictures are shown for each construct. Only the TgNCR1 construct that localizes to endo-lysosomes (TgNCR1-hNPC1) was able to facilitate cholesterol egress from these organelles. C. Quantitative measurement of filipin fluorescence levels associated with 2-2 mutant cells transfected with the indicated TgNCR1 constructs in comparison to wild-type CHO cells. Fluorescence intensity collected on 45–50 endo-lysosomes per condition was expressed as arbitrary values. Error bars indicate SEM, n = 3 (*, *P*<0.01 comparing TgNCR1-hNPC1-transfected cells with vector alone). D. TLC analysis of neutral (upper; FC, free cholesterol) and acidic (lower) fractions of cellular lipid extracts from CHO cells: wild-type or mutant transfected with either TgNCR1-hNPC1 or vector only. Positions of the standard lipids are indicated (left). After staining of the TLC plate, the band intensity of FC and GM1 (yellow asterisks) were measured by densitometry. Error bars indicate SEM, n = 3 (*, *P*<0.05; **, *P*<0.01 comparing TgNCR1-hNPC1-transfected cells with vector alone).

We used the somatic 2-2 mutant of CHO cells as a model of NPC1 mutant cells. These cells are characterized by the excessive accumulation of lipids including cholesterol in endocytic compartments [30]. We confirmed this phenotype by staining the 2-2 mutant cells with filipin, a fluorescent compound that binds to the β-hydroxyl group of sterols, and data revealed enlarged, filipin-positive structures corresponding to perinuclear lysosomes (Figure S4A). Our ultrastructural observations of sterol-overloaded lysosomes in filipin-treated 2-2 mutant cells illustrated the corrugated aspect of membranes as a result of the formation of sterol-filipin complexes (Figure S4B). The 2-2 mutant cells were transfected with p*myc*-TgNCR1-hNPC1 to assess sterol transport by filipin staining and fluorescence microscopy. We first confirmed the localization of the TgNCR1-hNPC1 fusion protein to perinuclear structures in 2-2 mutant cells (Figure 3A). Expression of the TgNCR1-hNPC1 fusion protein largely restored cholesterol clearance from endo-lysosomes (Figure 3B) as quantified by a ∼4-fold reduction in filipin intensity levels in endo-lysosomes of the transfected cells with TgNCR1-hNPC1 compared to mock-transfected cells (Figure 3C). Fluorescence levels in mutant cells expressing TgNCR1-hNPC1 were not significantly different from those associated with endo-lysosomes in wild-type cells (Figure 3C). Thin layer chromatography (TLC) analysis of a neutral lipid fraction of cellular extracts of TgNCR1-hNPC1-expressing mutant cells confirmed that the levels of free cholesterol have returned to normal (Figure 3D). No change has been observed in the levels of esters of cholesterol between wild-type cells, NPC1 mutant cells and mutant cells expressing TgNCR1-hNPC1 (Figure S5). The 2-2 mutant cells expressing TgNCR1 did not show any decrease in endo-lysosomal filipin staining, in accordance to the mislocalization of TgNCR1 in the ER. Negative controls for these experiments included mutant cells transfected with a truncated construct containing a short sequence of the C-terminus of TgNCR1 (from residues 978 to 1100) combined with the C-terminal end of hNPC1 containing the endosomal targeting motifs (Tr.TgNCR1-hNPC1). This chimeric construct localized both to endocytic compartments and the ER, and the filipin fluorescence levels in these cells were similar to those in TgNCR1-expressing cells.

The subcellular accumulation of sphingolipids, particularly gangliosides (e.g., GM1), is another characteristic of the NPC1 syndrome [31]. TLC analysis of an acidic lipid fraction from cellular extracts confirmed that these 2-2 mutant cells contained high levels of GM1, whereas in CHO wild-type cells the levels of this lipid were undetectable (Figure 3D). TgNCR1-hNPC1 expression in mutant cells caused an obvious decrease in the levels of GM1 compared to NPC1 mutant cells, and quantitative measurement indicated a reversion of the GM1 accumulation by ∼6-fold.

Altogether, these results suggest that, when provided with the correct localization motif for endocytic compartments, the TgNCR1constructs functions in a similar way as human NPC1 to promote LDL-cholesterol and GM1 release from endocytic organelles, which indicates a conserved activity of the two proteins.

### TgNCR1 localizes to the inner membrane complex in the parasite

Our data showed that in mammalian cells, the parasite and human NPC1 are interchangeable with respect to LDL-cholesterol transport from endocytic compartments. However, *Toxoplasma* never internalizes host cholesterol into endo-lysosomes, which predicts a function for TgNCR1 unrelated to exogenous sterol transport. This raises an intriguing issue about the biochemical function of TgNCR1 in *T. gondii*. As the first step towards understanding the molecular function of TgNCR1 in the parasite, we decided to examine its intracellular localization. For this purpose, a recombinant peptide corresponding to residues 162 to 501 of TgNCR1 was produced in *E. coli* to generate polyclonal antibodies in rabbits (Figure S6). Using anti-TgNCR1_162-501_ antibodies, immunofluorescence of intracellular wild-type parasites showed a diffuse peripheral staining along the parasite body, which seemed to be excluded from the apical and basal extremities of the parasite (Figure S7). Despite several attempts to purify these antibodies, a high level of fluorescence background remained both on the parasites and in host cells, which precluded the use of these antibodies for in-depth morphological studies.

**Figure 4:**
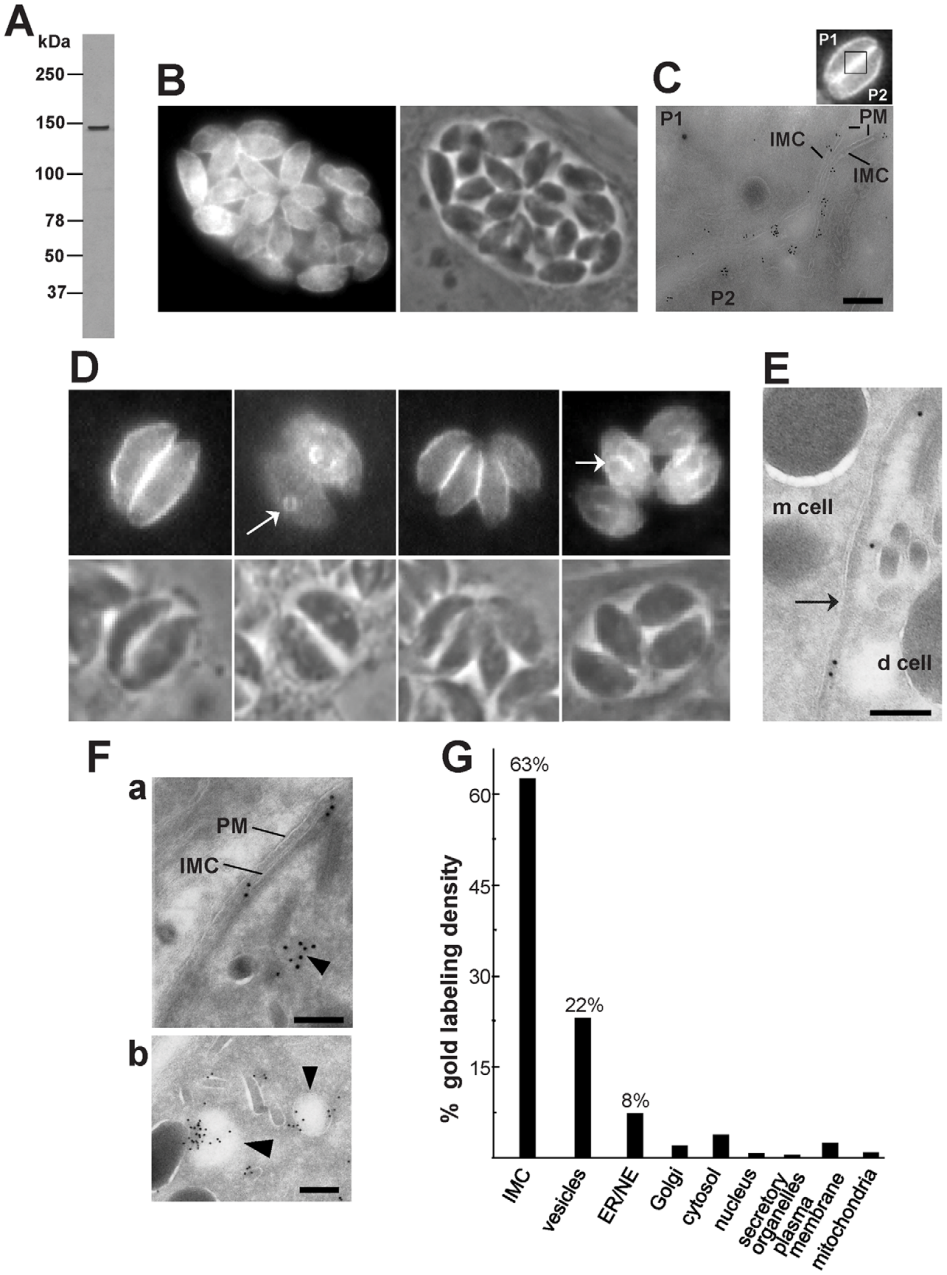
Expression and distribution of TgNCR1 in transgenic *T. gondii.* A. Immunoblot of *T. gondii* transfected with p*HA-TgNCR1*. Western blots were probed with anti-HA antibodies, revealing a single band at ∼140-kDa in transgenic parasites. No band was visible on immunoblots of wild-type parasites (not shown). B–C. Immunodetection of HA-TgNCR1 in transgenic parasites using anti-HA antibodies for IFA (in B) or immunogold staining (in C). HA-TgNCR1 is dominantly associated with the IMC. The image in C shows two adjacent parasites (P1 and P2, as represented in the demarcated area of the inset showing transgenic parasites stained by IFA) in a PV to emphasize the continuous staining of HA-TgNCR1 along the IMC. PM, plasma membrane. Bar is 150 nm. D–E. Immunodetection of HA-TgNCR1 in transgenic parasites using anti-HA antibodies for IFA (in D) or immunogold staining (E). HA-TgNCR1 is also located to the IMC of nascent daughter cells (d cell) as shown by arrows. m cell, mother cell. Bar is 150 nm. F. ImmunoEM on the transgenic parasites showing vesicular staining in addition to the IMC. Several vesicles close to the IMC were also decorated with gold particles (arrowheads in panels a and b). PM, plasma membrane. Bars are 150 nm. G. Stereological analysis of immunogold labeling demonstrating the preferential localization of HA-TgNCR1 on the IMC and neighboring vesicles in intracellular *T. gondii*. Density (gold particles per mm2) of labeled structures was determined from 30 cryosections. Percentage of individual intracellular compartment density was determined from the sum of gold density normalized for the variation in expression of TgNCR1. NE, nuclear envelope.

To circumvent this issue and have a better resolution of the TgNCR1 localization, we created a stable line of the parasite expressing, under the tubulin promoter, TgNCR1 with a HA tag at the N-terminus. Lysates of transgenic parasites were resolved in SDS-PAGE and immunoblot analysis using anti-HA antibodies, confirming the expression of exogenous HA-TgNCR1, which migrated as a single band at ∼140-kDa (Figure 4A). Immunofluorescence of intracellular HA-TgNCR1-expressing *T. gondii* showed a peripheral staining along the parasite body as observed on wild-type parasites labeled with anti-TgNCR1_162-501_ antibodies (Figure 4B). The apical and basal extremities of the parasite transfectants clearly excluded the staining, and this pattern is reminiscent of resident proteins of the inner membrane complex (IMC). EM observations reveal that the IMC is a continuous patchwork of flattened vesicular cisternae located beneath the plasma membrane and overlying the cytoskeletal network [32], [33]. The IMC arises from vesicles derived from the secretory pathway which flatten during parasite maturation to form large membranous sheets that envelop the parasite, leaving only a small gap at both parasite ends. TgNCR1 localization was further confirmed by immunoEM staining showing gold particles on the IMC (Figure 4C). *Toxoplasma* divides by endodyogeny, a mode of replication in which two daughter cells are produced within an intact mother parasite [34], [35]. A critical step in building daughter cells is the construction of their IMC, which forms a bud into which replicated organelles are packaged. Figure 4D reveals TgNCR1 labeling on the cone-shaped cistern forming nascent IMC, indicating the association of the protein with the daughter parasites at the onset of their formation. Immunogold staining confirmed an association of TgNCR1 with the IMC of nascent parasites (Figure 4E). Interestingly, the presence of gold particles was also visible on vesicles adjacent to the IMC (Figure 4F). Quantitative distribution of gold particles on various parasite compartments indicated that more than 80% of the gold staining was associated with both the IMC and vesicles, while the ER counted for 8% of the gold labeling density (Figure 4G). Finally to corroborate this IMC localization, we engineered two other constructs: TgNCR1, under the NTPase promoter, tagged at its N-terminus with the HA epitope and TgNCR1, under the tubulin promoter, tagged at its C-terminus with the HA epitope. Parasites were transiently transfected with each construct, and the original localization of TgNCR1 on the IMC was confirmed in these transgenic parasites expressing HA-TgNCR and TgNCR1-HA (Figure S8).

### Parasites expressing dominant negative mutants of TgNCR1 have no striking phenotype

As the second step towards clarifying the role of TgNCR1 in the parasite, we expressed in the wild-type *T. gondii* four different dominant negative mutants derived from HA epitope-tagged TgNCR1: HA-TgNCR1_D571N_, HA-TgNCR1_P913A_, HA-TgNCR1_I957T_ and HA-TgNCR1_L1100V_. These mutants are predicted to be dominant negative based on identified mutations in NPC1 patients [24]–[26]. When transiently transfected in the parasite for 16 h to 24 h, these constructs produced a signal that localized to the IMC/ER. The four dominant negative mutants were viable and gave no unusual phenotype, such as the abnormal accumulation of lipids based on TLC analysis, which precludes further functional studies (data not shown). It is possible that dominant negative mutants have lower expression levels than TgNCR1 wild-type protein, which renders the dominant-negative effect silent. Another possibility could be that the time-course for lipid accumulation takes longer than 24 h before giving a clear phenotype. While the expression level of the mutant proteins or the timing of the lipid accumulation phenotype may effect the lack of phenotype, it is also plausible that the amino acids mutated in the TgNCR1 sequence are not critical for its function in the parasite, which may be viewed as evidence that TgNCR1 plays distinct roles from human NPC1.

### Depletion of NCR1 does not affect parasite replication

**Figure 5:**
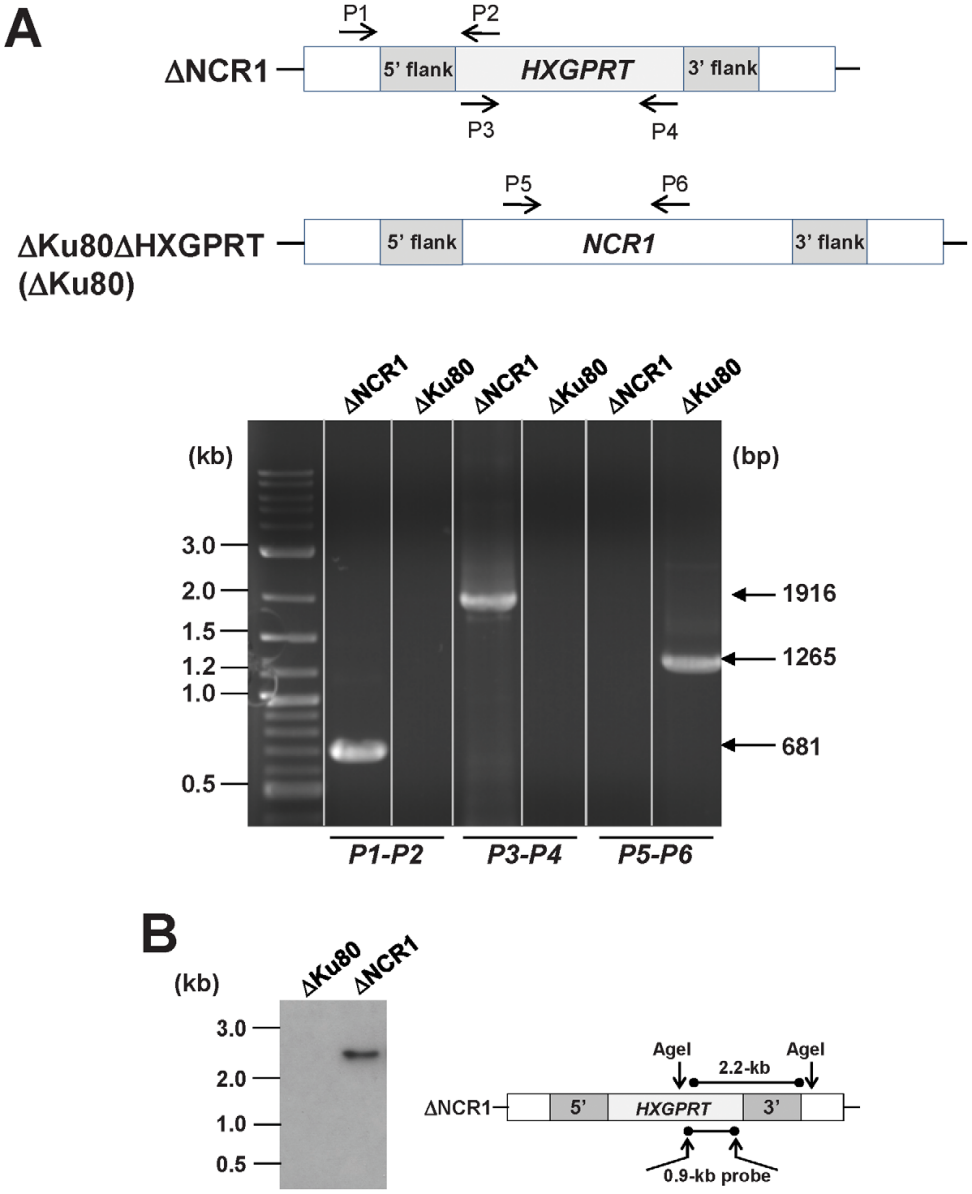
Validation of the targeted deletion of *NCR1*from the *Toxoplasma* genome. A. Top: schematic showing the three primer sets used to evaluate the parental strain ΔKu80 and ΔNCR1 for replacement of *NCR1* with the selectable marker *HXGPRT*; the presence or absence of *NCR1* and *HXGPRT* is shown on the gel (bottom). B. Southern blot of ΔKu80 and ΔNCR1 strains. Genomic DNA was isolated from each strain, digested with AgeI to excise a 2.2-kb fragment, run on an agarose gel, transferred to a nylon membrane and hybridized with a 0.9-kb probe that is part of the HXGPRT cassette.

As the third approach to study the function of TgNCR1 in *T. gondii*, we focused on genetically disrupting *NCR1* using a fusion PCR-based method to replace *NCR1* in the parasite genome with a selectable HXGPRT marker. Clones were tested by PCR and Southern blotting for the absence of *NCR1* and the presence of a single copy of the HXGPRT cassette (Figure 5 and Figure S9). Clones lacking *NCR1* were obtained, demonstrating that TgNCR1 was not necessary for the in vitro propagation of *T. gondii*. In fibroblasts, the ΔNCR1 strain formed large vacuoles that associated with host organelles, e.g., mitochondrion, as observed for wild-type parasites (Figure S10). The knockout parasites were organized in rosettes. During normal endodyogeny, the cytoplasm of the mother parasite is equally distributed between the daughter cells; a cleavage furrow is initiated at the anterior pole and extends between the daughters throughout the mother cell, leaving only a residual body at the posterior end connecting the two daughters. The residual body contains mother cell components that are not incorporated into the daughter parasites, and this structure usually disappears after the completion of parasite division. Interestingly, we observed that the progeny of TgNCR1-deficient parasites staid mostly connected by their posterior ends to a common and enlarged residual body. Similar prominent residual bodies were also apparent in light microscope images. Disruption of *TgNCR1* did not result in any obvious defect in parasite invasion or egress (data not shown).

**Figure 6:**
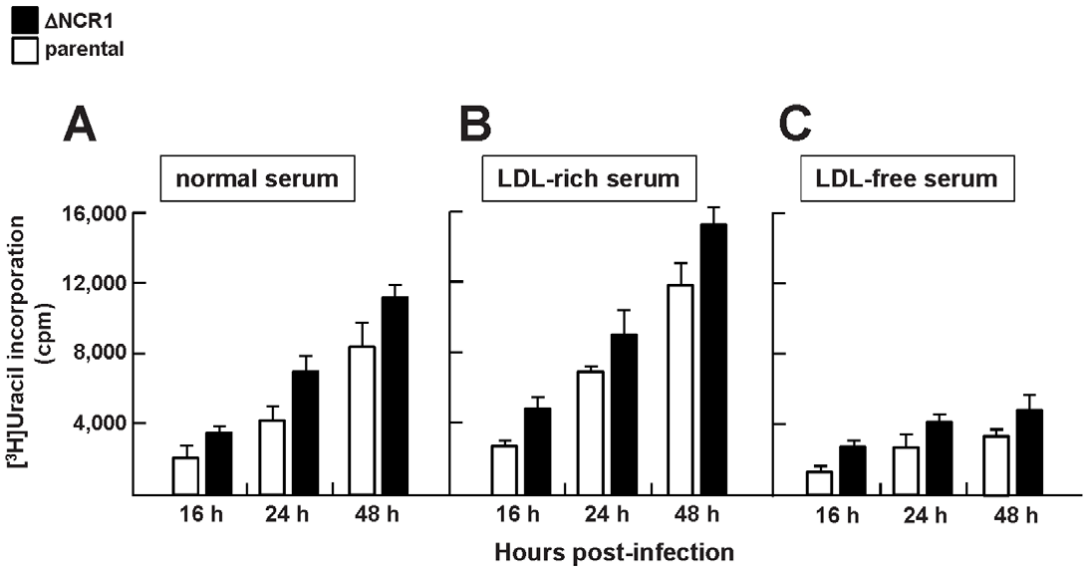
Growth features of TgNCR1-deficient parasites in vitro. Quantification of the replication rate of the ΔNCR1 and parental strains in medium with variable amounts of cholesterol. [^3^H]uracil incorporation was measured using HFF infected with the ΔNCR1 or parental strain for the indicated times in (A) medium containing 10% FBS (nominal LDL concentration: 0.1 mg/ml: normal serum), (B) medium containing 10% FBS containing 1 mg/ml LDL (LDL: rich serum) or (C) medium containing 10% FBS depleted of LDL (LDL-free serum). Values of uracil incorporation are means ± SEM from three separate experiments. Differences between values obtained for the two strains were statistically significant (*P*<0.05).

The growth rate of the ΔNCR1 strain in fibroblasts was quantified by uracil incorporation assays. Compared to the parental strain the (ΔKu80ΔHXGPRT or ΔK80), the ΔNCR1 strain incorporated significantly higher amounts of uracil (1.4-fold increase) at 16 h, 24 h and 48 h p.i. (Figure 6A). To verify the specificity of the mutant phenotype, we genetically complemented the ΔNCR1 strain with *TgNCR1*. TgNCR1-complemented clones were verified by PCR analyses (Figure S11). Values of uracil incorporation were comparable in the complemented and parental strains, corresponding to 2,392±504 cpm and 2,693±134 cpm, respectively, which indicates that the uracil incorporation defect of the ΔNCR1 strain was due to the deletion of *NCR1*.

### TgNCR1-deficient parasites are insensitive to excess cholesterol in the medium and contain normal amount of free cholesterol

We previously reported that *Toxoplasma* growth is directly dependent upon exogenously supplied cholesterol [2]. Mammalian NPC1 has a primordial function in exogenous sterol distribution throughout the cell [36]. TgNCR1-deficient parasites were then exposed to excess amounts of LDL-cholesterol to determine whether loss of *NCR1* has an effect on parasite viability as a result of the potential accumulation or mislocalization of cholesterol within mutant parasites. Results show that the addition of excess LDL in the medium, i.e. 10-times more than normal medium (10% FBS), did not affect the intravacuolar development of TgNCR1-deficient parasites (Figure 6B). A ∼150% increase in replication rate was observed for the ΔNCR1 strain compared to the parental strain grown in excess LDL, which parallels the increased replication rate of the ΔNCR1 strain in normal medium. We showed previously that exposure of *T. gondii* to a medium depleted in LDL results in a slowdown of parasite development as smaller vacuoles were observed compared to parasites developing in complete medium [2]. When TgNCR1-deficient parasites were incubated in the absence of LDL, they grew slower than in medium containing 10% FBS, but still showed a growth advantage over the parental strain grown in LDL-free medium (Figure 6C). Thus, the ΔNCR1 strain displays no significant change in sensitivity to extracellular cholesterol compared to parental parasites. This may predict a primary role for TgNCR1 that is unrelated to cholesterol transport.

**Figure 7:**
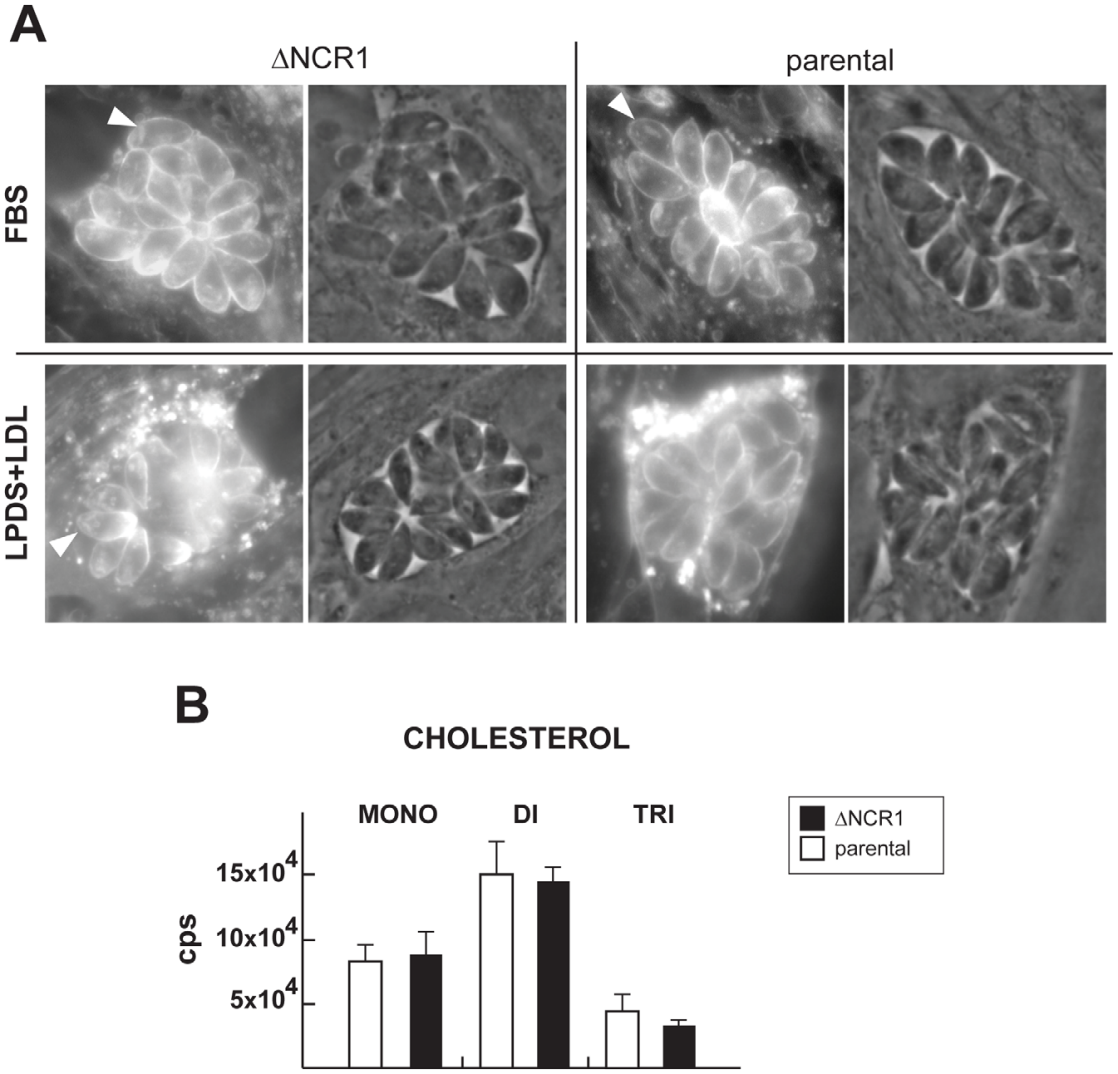
Cholesterol detection and quantification in TgNCR1-deficient parasites. A. Fluorescence microscopy using filipin to visualize the presence of cholesterol in the membrane of the ΔNCR1 and parental strains 24 h p.i. in either 10% FBS or 10% LDL-deficient serum (LPDS) containing 1 mg/ml LDL. No major difference in filipin staining is detectable. Arrowheads pinpoint parasite rhoptries revealed by filipin. B. Quantitative analysis of MRM spectra from the ΔNCR1 and parental strains showing relative levels of cholesterol monomers (mono), dimers (di) and trimers (tri). No significant differences could be observed between the two strains. Data are means ± SD from 3 independent populations of control and mutant parasites.

The NPC disease is characterized by lysosomal sequestration of endocytosed LDL-cholesterol, abnormal enrichment of unesterified cholesterol in *trans*-Golgi cisternae and anomalies in intracellular sterol trafficking [9]. We then monitored the cholesterol status in TgNCR1-deficient parasites compared to control parasites. In a first set of experiments, parasites were stained with filipin (Figure 7A). A fluorescence pattern was associated with the parasite's pellicle and apical rhoptries in both the ΔNCR1 and parental strains when cultivated in 10% FBS. When excess LDL was added to the medium, TgNCR1-deficient parasites did not show any major sites of cholesterol accumulation as is the case in NPC1-deficient mammalian cells. To validate our microscopic observations, we measured the levels of free cholesterol molecules, at monomeric and oligomeric states by mass spectrometry (Figure 7B). Our results established that the overall concentrations of monomeric, dimeric and trimeric cholesterol in ΔNCR1 were similar to the parental strain.

### TgNCR1-deficient parasites contain numerous lipid bodies in their cytoplasm and accumulate several species of cholesteryl esters

**Figure 8:**
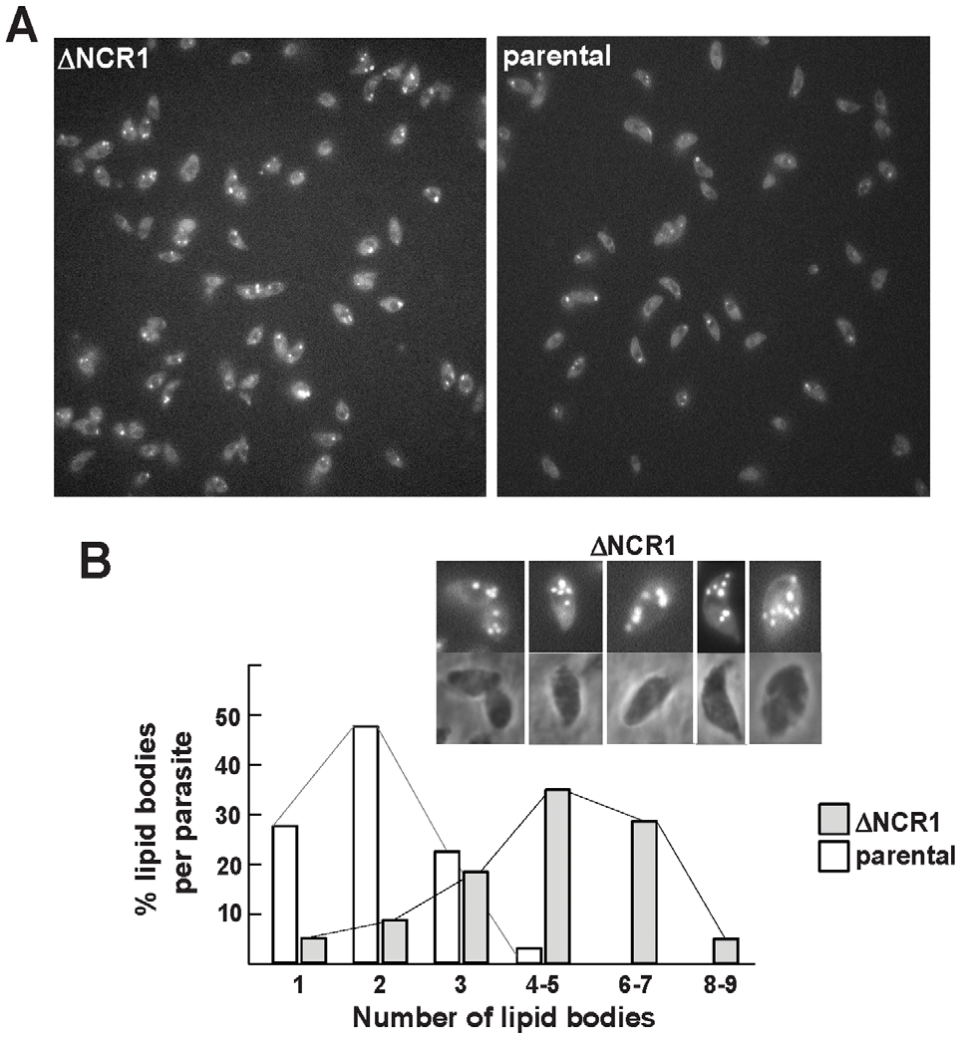
Production of lipid bodies by TgNCR1-deficient parasites. A. Fluorescence microscopy using Nile Red to stain lipid bodies in the ΔNCR1 and parental strains showing abundant fluorescent structures in TgNCR1-deficient parasites. B. Quantitative distribution of lipid bodies in the two parasite strains. Nile Red positive structures were counted, revealing greater number of lipid bodies in the ΔNCR1 strain. Insets show TgNCR1-deficient parasites with 4 to 9 large lipid bodies.

**Figure 9:**
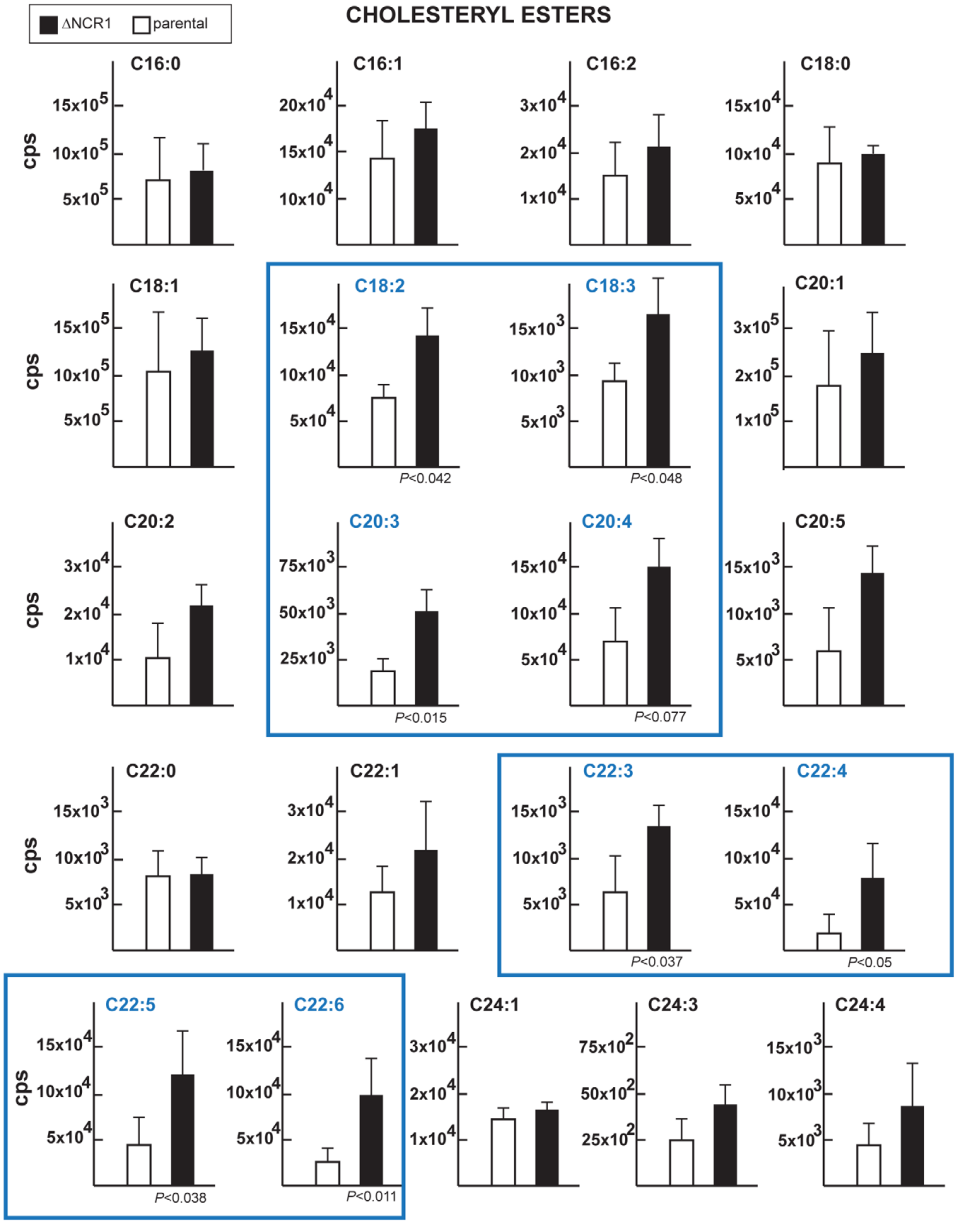
Content of cholesteryl esters in TgNCR1-deficient parasites. The levels of various species of cholesteryl esters were quantified from MRM spectra of the ΔNCR1 and parental strains. Statistically significant differences in the amounts of several cholesteryl esters were found in TgNCR1-deficient parasites. The y axes are in cps. Data are means ± SD from 3 independent populations from each strain.

To examine whether the lack of *NCR1* might impact the metabolism of lipids other than cholesterol, TgNCR1-deficient and control parasites were stained with Nile Red. This dye fluoresces when trapped inside lipid bodies allowing the examination of the cytosolic stores of neutral lipids in cells. Data show the presence of abundant lipid bodies in the ΔNCR1 strain, which were greater in size and number than those found in the parental strain (Figure 8A). The ΔNCR1 parasites could produce up to nine lipid bodies, with an average of five lipid bodies per cell whereas control parasites contained two lipid bodies per cell on average (Figure 8B). We previously described that *Toxoplasma* can store both esters of cholesterol [37] and triglycerides [38] in lipid bodies. The nature of neutral lipids accumulated in TgNCR1-deficient parasites was then investigated by mass spectrometry. Quantitative analysis of the cholesteryl ester species present in wild-type *Toxoplasma* was performed in detail (Table 1). As predicted by the detection of acyl-CoA:cholesterol acyltransferase (ACAT) activities in the parasite, cholesteryl esters were abundant in the parasite. Of the 21 molecular species detected in *Toxoplasma*, cholesteryl oleate C18:1 (42%) and palmitate C16:0 (26%) were the main esters as is the case in mammalian cells but the parasite also had uniquely large amounts of cholesteryl eicosanoate C20:1 (7%). In addition, the parasite contained to a lesser extent, cholesterol palmitoleate C16:1, stearate C18:0, linoleate C18:2, arachidonate C20:4 and some polyunsaturated C22 fatty acids. The other cholesteryl ester fatty acid species shown in Table 1 represented 3.5% of the total species. Interestingly, TgNCR1-deficient parasites showed an overall increase in the cholesteryl ester content, with the greatest accumulation of cholesteryl linoleate, arachidonate, C20:3 and some very-long-chain polyunsaturated fatty acids (Figure 9).

**Table 1:** Cholesteryl ester content and relative abundance in *Toxoplasma.*

SPECIES	% CHOLESTERYL ESTER SPECIES
**C18:1**	42.4
**C16:0**	25.5
**C20:1**	7.2
**C16:1**	5.5
**C18:0**	3.4
**C18:2**	3.0
**C20:4**	2.6
**C22:6**	1.9
**C22:5**	1.8
**C22:4**	1.7
**C20:3**	1.5
**C16:2**	0.6
**C24:1**	0.6
**C22:1**	0.5
**C20:2**	0.4
**C18:3**	0.3
**C22:0**	0.3
**C22:3**	0.3
**C24:4**	0.2
**C20:5**	0.2
**C24:3**	0.1

Quantitative analysis of MRM spectra from *T. gondii* showing the percent of species of cholesteryl esters.

Data have been collected from 3 different parasite preparations. Total area intensity is 2,651,000 cps (100%).

**Figure 10:**
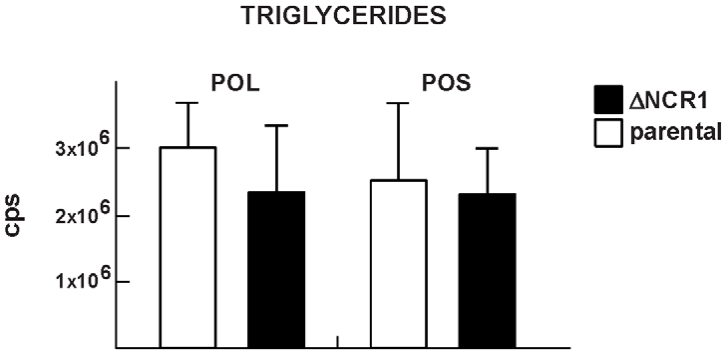
Content of triglycerides in TgNCR1-deficient parasites. Triglyceride levels were quantified from MRM spectra of the ΔNCR1 and the parental strains. Levels of two species of triglycerides: POS (palmitate:oleate:stearate) and POL (palmitate:oleate:linoleate) were similar in the two parasite populations. The y axes are in cps. Data are means ± SD from 3 independent populations from each strain.

In parallel, we examined the triglyceride content of the parasites (Figure 10). *Toxoplasma* contained pools of triglycerides with either palmitate:oleate:stearate or palmitate:oleate:linoleate, and these lipid species were also detected in TgNCR1-deficient parasites at similar levels. These results suggest that lipid bodies in the ΔNCR1 parasites are particularly enriched in cholesterol esters, as a result of an overproduction of these lipids and/or a dysregulation of the cholesterol cycle.

### TgNCR1-deficient parasites accumulate several species of very-long-chain fatty acid sphingolipids

**Figure 11:**
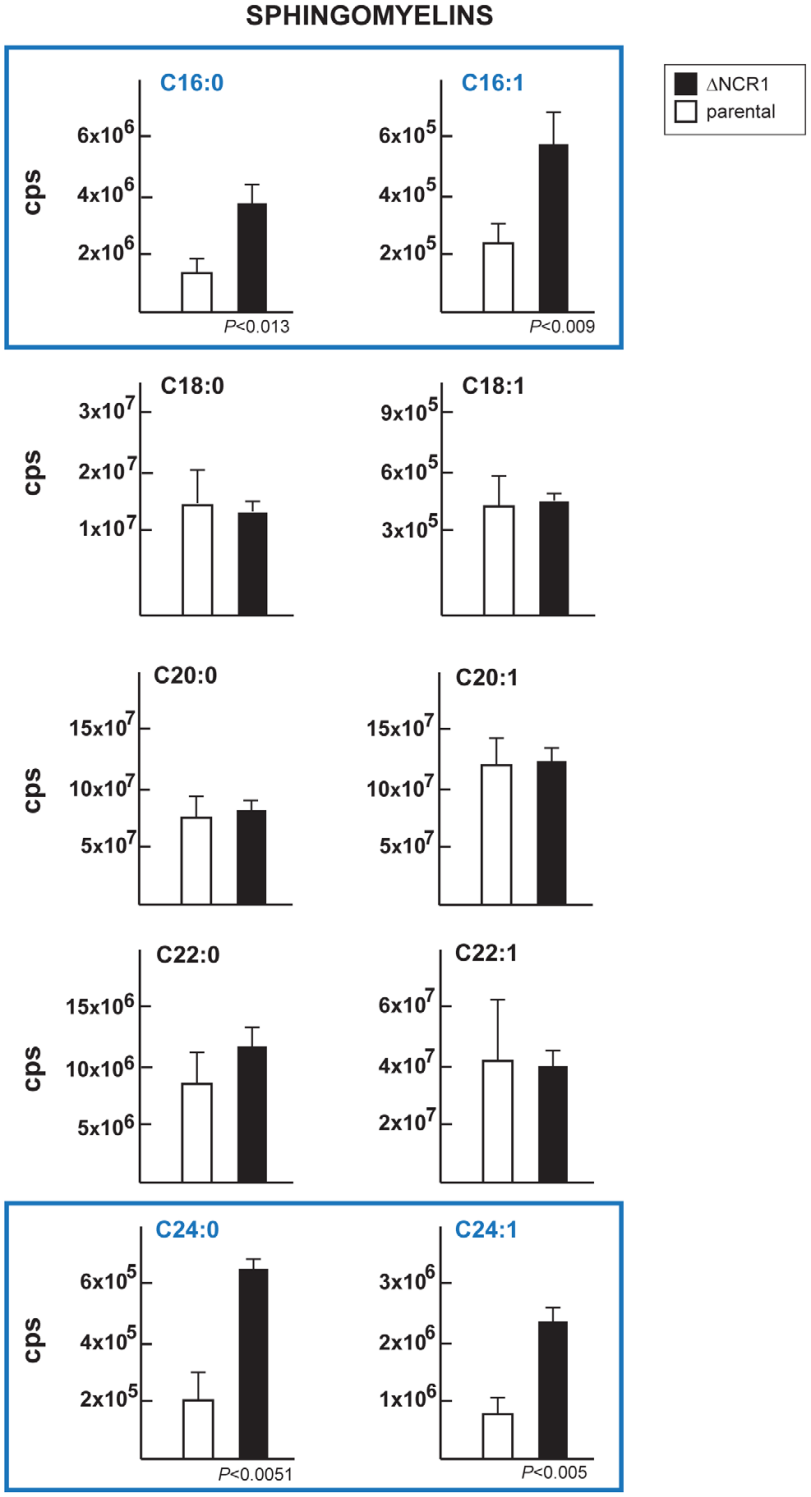
Content in sphingomyelins in TgNCR1-deficient parasites. The levels of several sphingomyelin species were determined by the quantitative analysis of MRM spectra from the ΔNCR1 and parental strains. Higher levels of C16 and C24 sphingomyelins were measured in TgNCR1-deficient parasites. Data are means ± SD from 3 independent populations from each strain.

**Figure 12:**
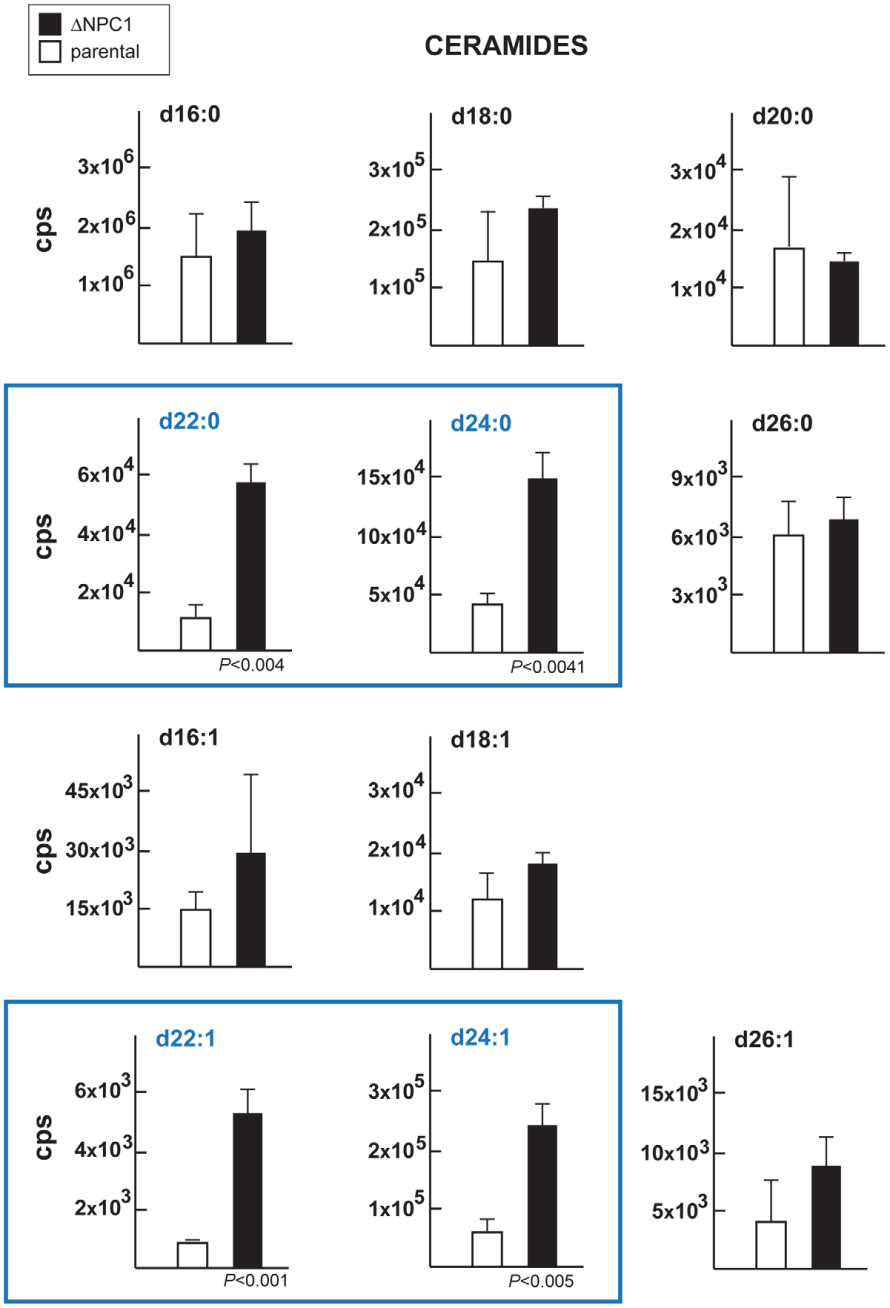
Content in ceramides in TgNCR1-deficient parasites. Levels of ceramide species were determined by the quantitative analysis if MRM spectra from the ΔNCR1 and parental strains. Higher levels of several very-long chain fatty acids ceramides in TgNCR1-deficient parasites were observed. The y axes are in cps. Data are means ± SD from 3 independent populations from each strain.

The NPC mutation has been also shown to impact the levels of sphingolipids [21], [39]. We then determined the status of sphingolipids in ΔNCR1 parasites. A previous quantitative study reported that sphingomyelins and ceramides represent 1.5 and 4.7% of total polar lipids respectively, in *T. gondii* [40]. We used quantitative mass spectrometry analyses to identify 10 different species of sphingomyelins (Table 2) and 11 species of ceramides (Table 3) in *Toxoplasma* controls. The main molecular species of sphingomyelins contained residues of arachidic acid C20:0 (44%), erucic acid C22:1 (28%) and stearic acid C18:0 (13%). Sphingomyelin species with eicosenoic acid C20:1 and behenic acid C22:0 were detected though in lesser abundance. Other species represented about 2% of the total sphingomyelins, with palmitic acid corresponding to 1%. The main species of ceramides in the parasite was palmitic acid (83%). Ceramides with stearic acid totaled 8% while the other ceramide species represented about 10%, with oleic acid less than 1%. Overall, the profiles of sphingomyelins and ceramides in *Toxoplasma* were quite different from those in mammalian cells in which oleic acid and palmitic acid are prominently represented. The sphingolipid profiles of TgNCR1-deficient parasites showed significant increases in both sphingomyelin and ceramide species containing lignoceric acid C24:0 and selacholeic acid C24:1 (Figures 11 and 12). In TgNCR1-deficient parasites, the ceramide C22:0 and the sphingomyelin containing palmitic acid and palmitoleic acid were also more abundant compared to the parental strain. Finally, to provide a complete overview of the lipid profiles in the ΔNCR1 strain, the status of phospholipids other than sphingomyelins was analyzed by quantitative mass spectrometry. It has been previously observed that the parasite phospholipid class distribution is significantly different from that of mammalian cells [40], [41]. Phosphatidylcholine is the most prevalent lipid, accounting for 61–75% of total phospholipids. The next most abundant lipids are phosphatidylethanolamine (10–16%), phosphatidylinositol (3.6–7.5%) and phosphatidylserine (5.6–6%). Data showed no difference in the levels of phosphatidylcholine and phosphatidylinositol between the ΔNCR1 and parental strains (Table S1). Only very few species of phosphatidylethanolamine and phosphatidylserine were significantly higher (up to a 3-fold increase) in TgNCR1-deficient parasites as compared to controls. Altogether, these data suggest that the main role of TgNCR1 in the parasite is in regulating the homeostasis of many different lipids.

**Table 2:** Sphingomyelin content and relative abundance in *Toxoplasma.*

SPECIES	% SPHINGOMYELIN SPECIES
**C20:0**	44.0
**C22:1**	28.0
**C18:0**	12.6
**C20:1**	7.8
**C22:0**	5.5
**C16:0**	1.0
**C24:1**	0.4
**C18:1**	0.4
**C16:1**	0.2
**C24:0**	0.1

Quantitative analysis of MRM spectra from *T. gondii* showing the percent of species of sphingomyelins.

Data have been collected from 3 different parasite preparations. Total area intensity is 158,990,000 cps (100%).

**Table 3:** Ceramide content and relative abundance in *Toxoplasma.*

SPECIES	% CERAMIDE SPECIES
**d16:0**	82.9
**d18:0**	8.0
**d24:1**	3.3
**d24:0**	2.2
**d16:1**	0.9
**d20:0**	0.8
**d18:1**	0.7
**d20:0**	0.6
**d26:0**	0.3
**d26:1**	0.2
**d22:1**	0.1

Quantitative analysis of MRM spectra from *T. gondii* showing the percent of species of ceramides.

Data have been collected from 3 different parasite preparations. Total area intensity is 1,808,300 cps (100%).

### TgNCR1-deficient parasites shift to the endopolygeny replication mode

**Figure 13:**
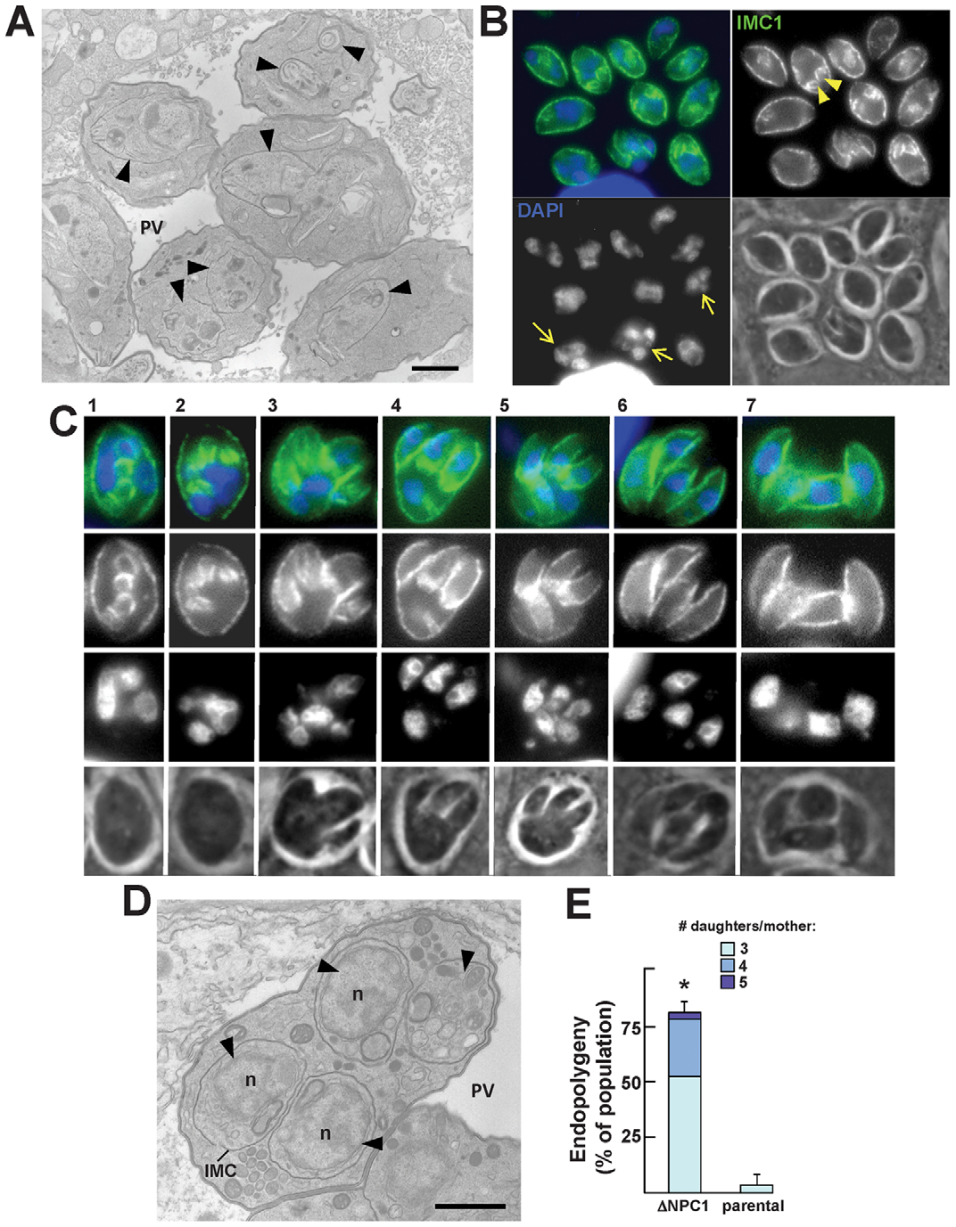
Peculiarities of replication of TgNCR1-deficient parasites. A. Transmission EM of TgNCR1-deficient parasites infecting HFF for 24 h. A. The PV contains many daughter parasites in formation (arrowheads). Replicating parasites were frequently observed for the ΔNCR1 strain. Bar is 1 µm. B–C. IFA on ΔNCR1 parasites infecting HFF for 24 h stained with antibodies against IMC1 as a marker for daughter buds (arrowheads in B). Parasites lacking NCR1 also assemble >2 daughters per round of division (arrows in B). C illustrates a parasite assembling four daughters. The process progresses from stage 1 (appearance of cone-shaped cisterns within the cytoplasm) to stage 7 (emergence of fully-formed daughters). Intermediate stages show that the mother cell has divided its nucleus and is budding new daughters around each separate nucleus (stained with DAPI). D. Transmission EM of a mutant parasite as described in panel A showing the formation of four daughters (arrowheads) within the mother cell. Each nascent daughter is delineated by its IMC. n, nucleus. Bar is 1 µm. E. Quantification of the >2 daughter phenotype in the ΔNCR1 and parental strains. Parasites undergoing endopolygeny were counted based on their DAPI-stained nuclei number and scored for the percentage of vacuoles in which parasites were assembling >2 daughters. Most PV contained parasites assembling 3 or 4 daughters in the ΔNCR1 strain. Values represent means ± S.D., n = 4 (*, *P*<0.0005).

We next examined the morphology of the TgNCR1-deficient parasites at the ultrastructural level. Astonishingly, nearly all EM sections demonstrated profiles of dividing parasites, indicating that these knockout parasites relentlessly underwent endodyogeny (Figure S10). While images capturing parasite endodyogeny can also be observed in wild-type parasites, it is at a much lower frequency. A representative PV containing many dividing TgNCR1-deficient parasites is shown in panel A of Figure 13, in which nascent daughter cells were visible within the cytoplasm of each mother cell. The sequential steps in parasite endodyogeny were then analyzed in detail (Figure S12). At the onset of daughter cell formation, the cytoplasm of the mother cell contained one or two thin, and either elongated (Figure S12, panel a) or horseshoe-shaped structures (Figure S12, panel b) that had the same electron-density as the IMC beneath the mother plasma membrane (Figure S12, panel a). These tubules likely represent the initial pieces of the new IMC that will assemble to provide the cytoskeletal scaffolding of developing daughter cells. As endodyogeny progresses, the nascent IMC extended in size and enveloped the dividing organelles, e.g., nucleus (Figure S12, panel c), Golgi and apicoplast (Figure S12, panel d), and the organelles produced de novo, e.g., rhoptries (Figure S12, panel Bd-e) and micronemes (Figure S12, panel e). The plasmalemma then invaginated around the daughters. Concomitant to the emergence of daughter parasites with an intact pellicle, the mother mitochondrial network moved to the posterior end of nascent parasites, bifurcated and entered the daughter scaffolds (Figure S12, panels f and g). These EM observations did not reveal any obvious defects in organellar partioning in daughters lacking *NCR1*, based on studies tracking fluorescent organelles in wild-type parasites by time-lapse microscopy. This suggests that the process of division of the ΔNCR1 strain occurs in a similar fashion as found for wild-type parasites.

The frequency of multiple daughter formation is usually rare in wild-type parasites and is typically in the range of 0.5 to 3% [42], [43]. Interestingly, we observed a significant high number of cases in which more than two daughters were formed within a single mother, as illustrated by immunofluorescence microscopy using the protein marker IMC1 to observe the IMC of nascent daughters within the mother cells (Figure 13B). We could observe the different steps of the construction of the multiple daughters per mother cell (Figure 13C). The budding seemed synchronized, and the time for multiple daughter formation did not reveal and delay compared to parasites undergoing normal binary division in the same vacuole. Transmission EM analysis also confirmed the presence of several individualized nuclei encircled by the IMC as a sign of assembly of new parasites (Figure 13D). To quantify this growth defect, we stained the ΔNCR1 and parental strain with DAPI and IMC1 and counted the PV containing parasites producing more than 2 daughters per mother (Figure 13E). Data show a dramatic increase in the number of endopolygeny events in the mutants with some mother parasites assembling as many as five daughters at the same time.

### TgNCR1-deficient parasites have an unusual cell cycle with a long S phase and short G_1_ phase

**Figure 14:**
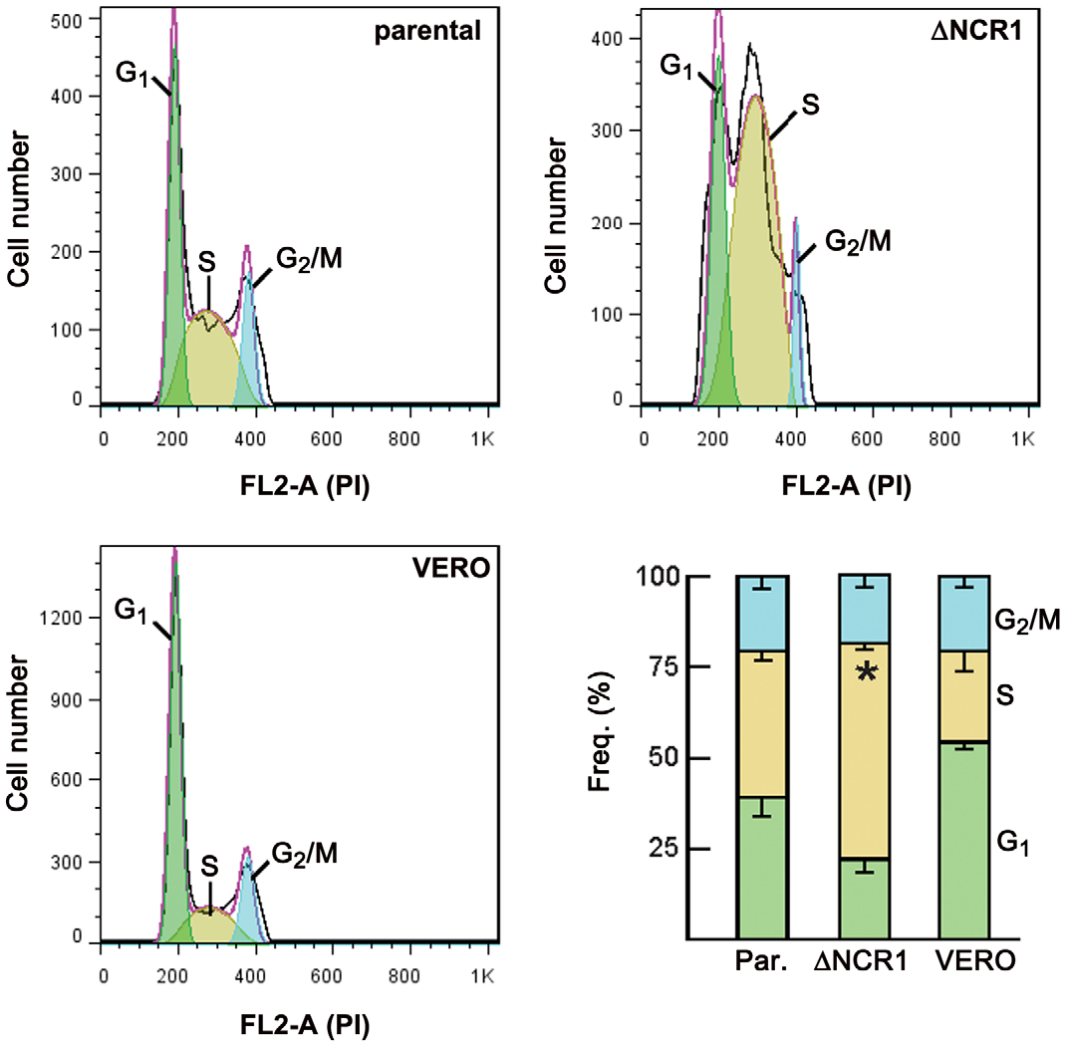
Cell cycle analysis of the ΔNCR1 and parental strains. Flow cytometry-based DNA content measurements of the ΔNPC1 strain in comparison with the parental strain and uninfected VERO cells as controls was performed at 15 h p.i. Isolated parasites from both strains and uninfected cells were fixed in ethanol and stained with the DNA-intercalating fluorescent dye propidium iodide (PI). The histograms show characteristic DNA distribution patterns in the three major phases of the cell cycle for each condition. The bar graph demonstrates the percentages of cells in each phase of the cell cycle. Data are means ± SD of three independent assays. The percentage of TgNCR1-deficient parasites accumulated in the S phase was significantly higher than that calculated for control parasites (*, *P*<0.005).

*Toxoplasma* tachyzoite replication differs from the classic animal cell cycle as they divide using a three-phase cycle (G2 may be short or missing) with the G1 interphase period comprising 40–60% of the parasite's doubling time [reviewed in 35]. S phase distributions in *T. gondii* are also peculiar, with late S phase parasites (1.8 N) being more numerous than early S phase parasites. Internal daughter budding appears to initiate in late S phase. We performed flow cytometry analysis on the ΔNCR1 strain to examine the cell cycle profiles of these mutants, in comparison with control parasites and VERO cells from which these parasites have been isolated (Figure 14). Data show that the percentages of parasites in G_2_/M phase were comparable between the mutant and parental strains and represented consistently a fraction between ∼10–12% of the total population. However, the average percentage of TgNCR1-deficient-parasites in S phase was significantly higher than for control parasites (about 61% vs. 41%). Consequently, the population of parasite mutants during the G_1_ phase corresponded to ∼24% only.

### The phenotype of TgNCR1-deficient parasites is associated with long PV membranous extensions

We next wanted to focus our attention to the PV of the ΔNCR1 strain to detect any peculiar morphological changes compared to the parental strain. A feature commonly shared by many intravacuolar pathogens is the formation of membranous tubules or fibers that extend away from their vacuole to facilitate their replication [44]–[46]. These filamentous structures contain many pathogen-derived proteins. *Toxoplasma* also forms long tubules extending from the PV membrane containing proteins released from dense granules, e.g., GRA7, GRA10, GRA14 proteins [3], [45] (Figure S13). We then examined the morphology of the PV membrane, with special emphasis on the tubules emanating from this membrane by fluorescence microscopy.

**Figure 15:**
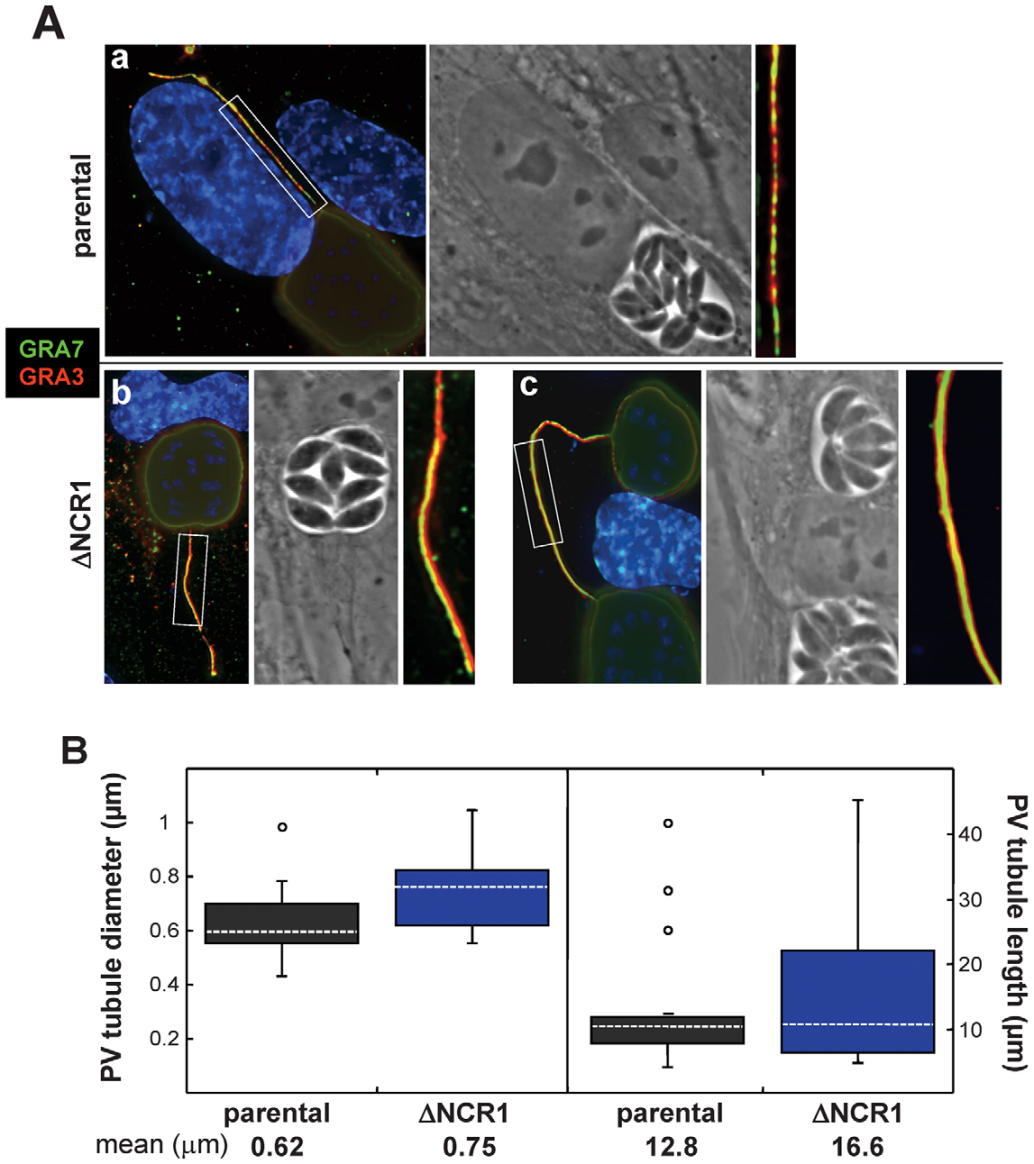
Characteristics of PV membranous extensions formed by the ΔNCR1 and parental strains. A. Double IFA on HFF infected with either the ΔNCR1 or parental strain 24 h p.i. using anti-GRA3 and anti-GRA7 antibodies revealing more intense fluorescence on extensions from the PV membrane of the TgNCR1-deficient parasites (insets). B. Quantitative analysis of PV membranous extensions for both strains. HFF cells were infected with either strain for 36 h. The samples were fixed and stained using anti-GRA3 antibodies. Serial optical z-sections of about 50 PV randomly selected were obtained from each strain using fluorescent microscopy. The diameter and length of the PV tubules were measured using Volocity software. Values shown are means ± SD of 3 independent infected monolayers for each condition (*P* values are 0.008 and 0.334 for the diameter and the length, respectively).

Fibroblasts were infected with the ΔNCR1 or parental strain for 36 h for double IFA to detect GRA3 and GRA7 on the PV membrane (Figure 15A). Both strains form long extensions originating from the PV. In multi-infected cells, some of these tubules were connecting two PV. The fluorescent signal for GRA3 and GRA7 appeared much brighter and homogenous for the extensions of the PV containing TgNCR1-deficient parasites than those generated by the parental strain, suggestive of more robust extensions. Morphometric quantification of the diameter and length of PV membranous extensions confirmed that they were significantly thicker and longer for TgNCR1-deficient *Toxoplasma* (Figure 15B).

**Figure 16:**
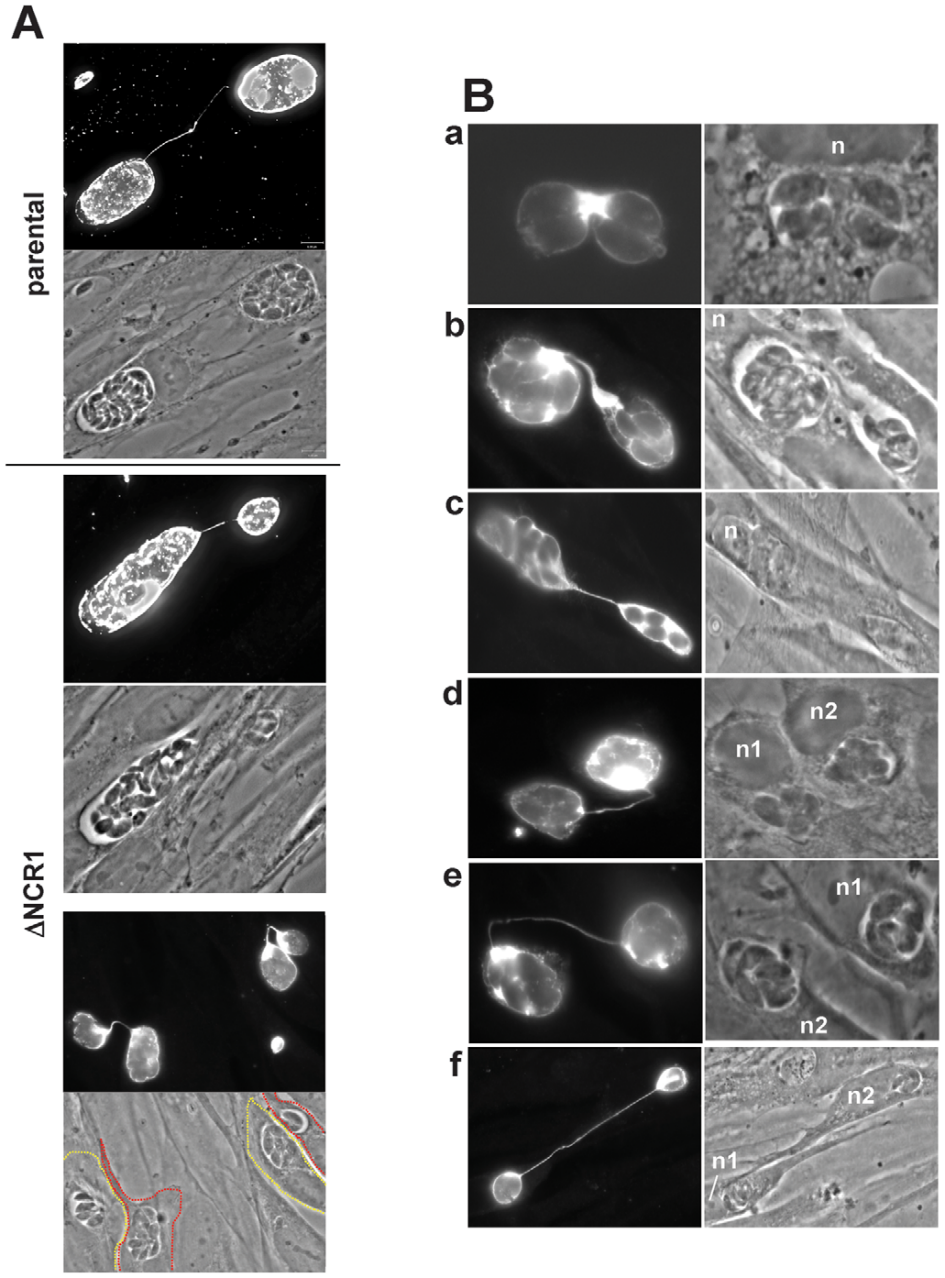
Detection of membranous connections between the PV of TgNCR1-deficient parasites in different cells. A. IFA on HFF infected with either the ΔNCR1 or parental strain 36 h p.i. using anti-GRA7 antibodies illustrating the connection of two PV in different cells mediated by membranous extensions. B. IFA on HFF infected with the ΔNCR1 strain 24 h p.i. using anti-GRA7 antibodies showing different views of PV connected to each other in the same cells (panels a–c) or neighboring cells (panels d–f). n, host nucleus.

Interestingly, we observed that several PV of TgNCR1-deficient parasites, although located to different host cells, were connected together by these PV membranous extensions (Figure 16A). In some occasions, two PV of the parental strain residing in adjacent cells were also seen joined together by PV tubules, suggesting that this phenotype plays a physiological role during parasite development. Close observations of the PV of TgNCR1-deficient parasites revealed that the closer two PV were to each other, the thicker the connecting tubules were (Figure 16B, panels a to c). A plausible scenario would be that the tubular structures bridging two PV may be generated consequently to the split of a PV into two vacuoles that stay connected by their PV membrane which elongates as the two PV separate from each other. Finally, we observed that these membranous connections between PV were maintained after host cell division, and dramatically increased in length as the two PV move apart (Figure 16B, panels d to e). The tubules originated from the PV membrane are likely made of lipids. The abundant and thick membranous extensions formed by TgNCR1-deficient parasites may suggest that these mutants may have an unusual membrane lipid biogenesis.

### Disruption of *NCR1* results in increase in virulence in mice

**Figure 17:**
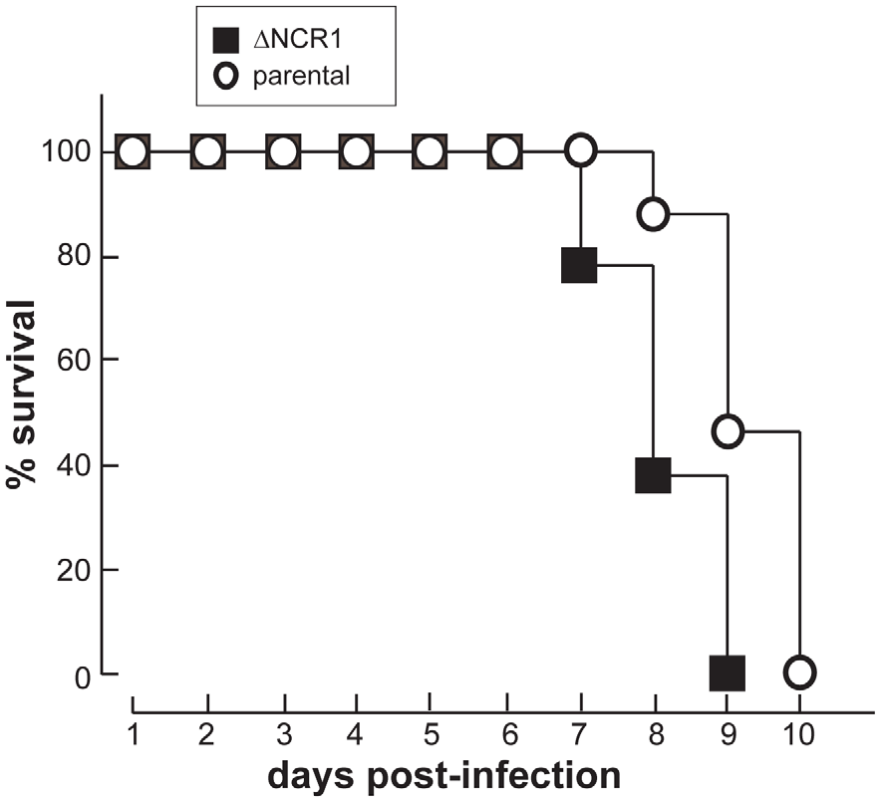
Growth features of TgNCR1-deficient parasites in vivo. Virulence assays of TgNCR1-deficient parasites. 5×10^4^ parasites of the ΔNCR1 or parental strains were intraperitoneally injected into four BALB/c mice per group. Data shown are means of 3 separate assays with <5% variation (S.D.).

It still remains unclear why TgNCR1-deficient parasites can generate more than two daughters from one mother cell. Nevertheless, if mutant daughter cells are viable and infective, the increase in their production may contribute to the more rapid expansion of the knockout population in vivo.The fast development of TgNCR1-deficient parasites in vitro prompted us to examine whether the ΔNCR1 strain is particularly virulent in mice. To examine the role of TgNCR1 in *Toxoplasma* infectivity, mice were infected with either the ΔNCR1 or parental strain after intraperitoneal injection, and mice survival was monitored daily. All mice infected with the parental strain died by day 10 (Figure 17). By comparison, mice parasitized with TgNCR1-deficient *T. gondii* showed higher susceptibility to infection as all mice systematically died one day earlier, as monitored in three independent trials. This suggests that TgNCR1-deficient parasites are slightly more virulent than control parasites.

## Discussion

As a starting point for studies on lipid transport and regulatory processes within the parasite, the aim of this work was to investigate the role of a unique SSD-containing protein, TgNCR1, expressed by *T. gondii*. The sequence of TgNCR1 predicts an uncharacterized Niemann-Pick, type C1-related protein with significant identity (37%) to human NPC1. The TgNCR1 sequence predicts a 1178-residue protein (131-kDa) with four potential N-glycosylation sites, and regions homologous to the Patched Homology Domain (PHD), the SSD and the sterol-binding domain (SBD) [5]. Hydropathy plots and topology predictions of TgNCR1 and human NPC1 suggest a striking conservation of the secondary structure between the two proteins, in that both have 11 to 12 transmembrane domains whose spacing is conserved. Interestingly, a *NPC1* homolog is present in several species of apicomplexan parasites and their predicted sequences are closely related to that of *TgNCR1*. Mutations in human NPC1 that cause NPC disease lay in residues conserved in TgNCR1. When expressed in mammalian cells with impaired NPC1 activity, TgNCR1 can restore the sterol and GM1 trafficking. Overall, despite the evolutionary distance of mammals and protozoa, human NPC1 and TgNCR1 are very functionally interchangeable at least for LDL-cholesterol and sphingolipid movement.

The ability of TgNCR1 to complement a mutant human NPC1 is *a priori* intriguing given that the core problem of the NPC disease is an accumulation of cholesterol and other lipids in certain endocytic organelles [9], [47]. *T. gondii* does not have a process of receptor-mediated uptake of sterols nor contains degradative lysosomes. In fact, though *T. gondii* is auxotrophic for cholesterol derived from plasma LDL [2], the parasite internalizes this lipid by using membrane translocators [5]. Nevertheless, the detection of cholesterol transport activity in NPC1-deficient cells expressing TgNCR1 implies that the parasite protein can bind cholesterol, perhaps by its putative SBD, and is maybe recognized by necessary accessory proteins in mammalian cells, e.g., NPC2 for cholesterol egress from endo-lysosomes. Though NPC2 proteins are highly conserved across genera that have clear NPC1 orthologs, a gene encoding a NPC2-like protein could not be identified in *T. gondii*. All of these intriguing observations predict that TgNCR1 would function in an unconventional way in *Toxoplasma*, likely distinct from a role in exogenous cholesterol transport. To explore the function of TgNCR1 in *Toxoplasma*, a parasite strain lacking the *NCR1* gene was generated. ΔNCR1 progeny are viable, indicating that *NCR1* is not required for replication. Altering the environmental cholesterol conditions by either starving *Toxoplasma*-infected cells for LDL or feeding infected cells with excess LDL, did not disturb the growth of knockout parasites differently than the parental population. The ΔNCR1 strain is not overloaded with free cholesterol, as are mutant mammalian NPC1 cells but instead abnormally accumulates sphingolipids and cholesteryl esters, and to a lesser extent, phosphatidylethanolamine and phosphatidylserine. Since NPC1 proteins universally function as lipid transporters, the observed accumulation of lipids in the ΔNPC1 strain is most likely the primary effect resulting from the loss of *NCR1*. Consequently, TgNCR1 seems to be involved in controlling the intracellular levels of specific lipids in the parasite. The yeast NPC homolog (NCR1), which is localized to the vacuole, functions in sphingolipid recycling and not in sterol transport even though yeast NCR1 complements the loss of NPC1 in mammalian cells [21]. NPC1 proteins also bear intriguing similarities to the Resistance-Nodulation Division (RND) system of bacterial permeases, which transport acriflavine and fatty acids [48]. Heterologous expression of RND from *Escherichia coli* in human NPC1-deficient fibroblasts results in the accumulation of acriflavine and fatty acids, but not the restoration of the NPC1 sterol accumulation phenotype. As *Toxoplasma*, yeast and bacteria do not internalize sterols from their external environment by endocytosis, our study here widen the concept proposed by SL Sturley [16] that in cells that do not ingest exogenous cholesterol in endocytic organelles, the NPC1 proteins do not primarily function as a cholesterol sensor/transporter, but as a regulator of the movement of other lipophilic substrates including sphingolipids. In this case, the role of NPC1 in higher eukaryotic cells as a cholesterol transporter might have arisen during evolution concomitantly to the development of receptor-mediated pathways for sterol uptake. Nevertheless, it remains possible that the accumulation of cholesterol in mammalian late endosomes may not be the primary cause of NPC disease but a secondary effect as a consequence of impaired transport of other metabolites aside from sterols.

The abnormal lipid accumulation resulting from the loss of *NCR1* in *T. gondii* leads to secondary defects including the stimulation of daughter formation, virulence, membrane biosynthesis and lipid body biogenesis. Mutants produce more progeny parasites than controls. The accumulation of mutant parasites in S phase seems attributable to the absence of *NPC1* since this phenomenon has not been observed in the parental strain or wild-type population of *T. gondii*. This feature may be linked to the propensity of TgNCR1-deficient parasites to form multibuds with multiple nuclear divisions between rounds of S and G_2_/M phase. During endopolygeny, the parasites reinitiate DNA synthesis bypassing G1 checkpoints but then re-coordinate the cell cycle as they reach the next mitosis. By analogy, mammalian tumor cells with high proliferation rate, e.g., HeLa cells are also characterized by cell cycle alterations with the observed accumulation of cells in S phase and reduction of cells in G_1_ phase. Such a deregulated cell cycle in cancer cells is mainly due to defective genome-integrity checkpoints, leading to abnormal DNA content. Several cell cycle checkpoints with controllers of cyclin-Cdk activity (G_1_, START (G_1_/S) checkpoints and mitotic controls) have been characterized in *Toxoplasma* [35]. However, the synchronization of multibud formation in the ΔNCR1 strain precludes the idea that these parasites escape from the regulatory systems of checkpoints, which complete mitosis in synchrony and control the quality of progeny.

TgNCR1 localizes to the IMC, a unique cortical system of membrane cisternae connected to the plasma membrane. As in mammalian cells, most of the parasite's cholesterol is concentrated at the plasma membrane [49]. However, it has also been documented that the IMC contains cholesterol-rich domains in which the parasite myosin motor machinery is immobilized [50]. These domains are critical for parasite gliding motility and attachment to a substrate. The location of TgNCR1 to the parasite IMC underlines the possibility that TgNCR1 may act as a catalyst to assemble cholesterol-rich. The highly dynamic properties of the IMC during parasite replication could facilitate the trafficking and mobilization of lipids necessary for daughter cell formation. Disruption of *NCR1* results in endopolygeny. This suggests that the function of TgNCR1 may be relevant in some way for coordinating normal progeny number and assembly, for example by delivering appropriate amount of lipids for membrane/organelle biosynthesis. It has been reported that genetic factors or local conditions increase the tendency to form multiple daughters [42]. For example, the ISP1 protein, which is located to the apical portion of the IMC, is involved in the targeting of many proteins to the IMC and seems to play a role in synchronizing endodyogeny [51]. Interestingly, *T. gondii* lacking ISP1 also assemble more than two siblings per mother cell. Observations based on TgNCR1 and ISP1 mutants suggest an important contribution of the IMC to the regulation of parasite division pathways, and thus to the control of infectivity.

Deletion of TgNCR1 from parasites leads to an increase in virulence as mice infected by the mutant strain succumb slightly faster than mice infected with control parasites. The fitness of TgNCR1-deficient parasites in vivo may be due to their higher propensity to produce multiple daughters, and therefore to disseminate faster within the host. Alternatively, the high content of sphingolipids inTgNCR1-deficient parasites may lead to increased virulence for several reasons: i) these lipids may play an important role to promote the parasite's intracellular development, e.g., by the induction of long PV membranous extensions that facilitate the parasite colonization of host cells; ii) the sphingolipids may act as immunomodulators during the progression of the infection; and iii) these lipids may stimulate the expression of virulence factors. First, the excess sphingolipids may promote intracellular parasite development as in mammalian cells it is known that sphingolipids are implicated in the regulation of cell growth and differentiation via DNA stimulation [52]. These lipids act as intracellular second messengers, and when added exogenously, they elicit diverse cellular responses related to cell division. Second, in different mammalian cell types, sphingolipids and their derivatives are involved in inflammation and can initiate parts of the inflammatory process by activating pro-inflammatory transcription factors [53]. It is known that the overproduction of pro-inflammatory cytokines, particularly IL-12, INF-γ, and TNF-α by mice infected with virulent strains of *T. gondii* contributes to the animal death due to apoptosis in vital organs [54]. Lastly, the mutant parasites may use its sphingolipids as part of its signaling machinery to stimulate the expression of virulence factors secreted into the host cell, e.g. ROP proteins that control the host transcriptional responses by subverting STAT3 and STAT6 signaling [55].

Though there are widespread disruptions in lipid metabolism in TgNCR1-deficient parasites, the observed increase in lipid body biogenesis may represent a bypass pathway utilized by the parasite to repress the consequences of the loss of *NCR1*. Bypass pathways have been described in mammalian cells expressing a mutated NPC1 protein. For instance, chemicals that activate protein kinase C or elevate cytosolic calcium can revert the NPC phenotype [47]. In addition, overexpression of Rab7 (involved in early/late endosome to lysosome transport) or Rab9 (involved in late endosome to Golgi transport) results in a reduction in the amount of cholesterol stored in late endosomes. Overexpression of Rab7 or Rab9 also induces an increase in Nile Red staining, which presumably reflects an increased storage of neutral lipids, including cholesterol esters in lipid bodies. The synthesis of cholesteryl esters, which have lower biological toxicity than free sterols, provides an efficient and readily accessible sink for cholesterol, and this process can then compensate for the loss of NPC1 activity. By analogy, the accumulation of additional lipid bodies and the bulky production of cholesteryl esters in TgNCR1-deficient parasites may be viewed as a compensatory mechanism activated by the mutant parasites to circumvent the lipotoxic effects of free cholesterol accumulation. Although we do not know whether there is transient accumulation of free cholesterol in mutant parasites, the high number of cholesteryl ester-containing lipid bodies would implicate TgNCR1 in cholesterol movement e.g., delivery of cholesterol to organelles. In fact, the trafficking of cholesterol to large-size lipid bodies has been observed in human NPC fibroblasts [56]. The enlargement of lipid bodies is caused by the abnormal fusion of lipid bodies with one another, but upon correction of the NPC phenotype by ectopic expression of NPC1, cholesterol is targeted to small-sized lipid bodies. It has been proposed that the activity of NPC1 resides in the maintenance of normal sized lipid bodies, possibly by preventing their fusion. *T. gondii* contains two ACAT enzymes located to the ER, which are both essential for parasite development [37; Lige and Coppens, data in preparation]. Inventories of cholesteryl esters produced by *Toxoplasma* reveal a predominant content of cholesteryl oleate and palmitate (∼70%) as well as other esters with polyunsaturated fatty acids that are rarely found in mammalian cells. An overall increase of ∼30% in cholesteryl esters is observed in TgNCR1-deficient *Toxoplasma*, reflecting that the cycle of esterification/hydrolysis of cholesterol esters is severely dysregulated in mutant parasites. This phenotype may result in an impairment of cholesteryl ester hydrolysis to free cholesterol. The *Toxoplasma* genome contains a gene coding for a cholesteryl ester hydrolase homolog (TgME49_038200) that can be involved in this function. Alternatively, ACAT activities can be stimulated in mutant parasites in response to accumulated free cholesterol. Concomitant to the increase in cholesteryl ester content, the number and size of lipid bodies are dramatically enhanced in mutant parasites, probably to collect excess cholesteryl esters. While TgNCR1 has never been detected on parasite lipid bodies, the accumulation of lipid bodies and cholesteryl esters in the ΔNCR1 strain suggest that TgNCR1 may regulate the cycles of cholesterol esterification and lipid body content.

Lipidomic analyses reveal that *Toxoplasma* contains more than twenty different species of sphingolipids consisting of both saturated and unsaturated fatty acids. The origin of sphingolipids in the parasite remains unclear. Evidence for de novo sphingolipid synthesis by *Toxoplasma* is based on the indirect effect of drugs known to block sphingolipid biosynthesis in mammalian cells or fungi but their enzymatic targets have not been identified in the parasite [57]. Other studies support the active scavenging of host cell-derived ceramides by *Toxoplasma* [58; Romano and Coppens, data in preparation]. Like in NPC1-deficient mammalian cells, the homeostasis of sphingolipids is altered in TgNCR1-deficientparasites, with a selective accumulation of several species of very-long-chain fatty acids. A few species of phosphatidylethanolamine and phosphatidylserine also show increased levels in the mutant parasites. The cause of this accumulation is unknown. One possible explanation may be linked to the multiple biosynthetic pathways of serine. Besides being the precursor of ceramides, serine is involved in the formation of phosphatidylserine and phosphatidylethanolamine through ethanolamine synthesis. Increases in the levels of phosphatidylethanolamine and phosphatidylserine may be due to the enhanced synthesis of these phospholipids via the diversion of serine from sphingolipid pathways. The dysregulation of the sphingolipid levels in the ΔNCR1 strain may have an indirect effect on the metabolism of phospholipids, which use the same precursor. Interestingly, an accumulation of other phospholipids, e.g., phosphatidylcholine has been reported in NPC1-deficient hepatocytes though the mechanism has not been explored [59].

In mammalian cells, sphingolipid metabolism is closely coordinated with that of sterols as these two lipids associate physically and there is considerable cross-talk between their metabolic pathways [60]. Among the cooperative activities of cholesterol and sphingolipids is the nucleation of lipid microdomains. Imbalance in either of these lipids results in disease pathology. It is possible that perturbations in sphingolipid levels in the ΔNCR1 mutant parasites may be related to changes in cholesterol homeostasis, either as feed-forward or feed-backward sequelae of cholesterol imbalance. In addition, the accumulation of sphingolipids in the ΔNCR1 strain may be due to impaired catabolism, altered vesicular transport of these lipids and/or of cholesterol, or blockade of sphingolipid export. In eukaryotic cells, sphingolipids and their metabolites play key roles both as structural components of membranes and signaling molecules that mediate responses to physiologic cues and stresses [61]. Sphingolipids are particularly abundant on the extracellular face of the plasma membrane, where they participate in cell-cell communication and host-pathogen interactions [62], [63]. The structural features of sphingolipids can influence the order of the lipid phase, and impact membrane curvature and thickness [64], [65]. The distribution, function and regulation of sphingolipids in *Toxoplasma* as well as the contributions of these lipids to parasite development are unknown. Long extensions derived from the PV membrane are observed in mutant parasites. It is then tempting to propose that these extensions may result from the selective incorporation of sphingolipids, i.e. sphingomyelins in this membrane, which contributes to the stability of the extensions. It might be of interest to compare the *Toxoplasma* PV tubules extending into the host cytoplasm with the tubovesicular network derived from the PV of intraerythrocytic *Plasmodium* that also pervades the host cytosol. Interestingly, the biosynthesis of sphingolipids in the malaria parasite is an import feature of this tubovesicular network and its membranes accumulate specific sphingolipids including sphingomyelin [66], [67]. The physiological role of such extensions in *Toxoplasma* is still unknown. They may participate in host cell remodeling by deploying parasite proteins into the host cell and recruiting host organelles to the PV. These extensions may also serve as a route for exchange of material between two PV. The intracellular bacterium *Chlamydia trachomatis* forms long fibers extending from the inclusion membrane into the host cytosol [37], and it has been proposed that the fibers play a role in facilitating the generation of new infective vacuoles, and therefore promoting bacteria replication in the host cell. Some PV membranous extensions can also connect two PV located in different host cells and our preliminary studies reveal the presence of these extensions inside nanotubules connecting two mammalian cells (Romano and Coppens, data in preparation). This feature may represent an efficient means for *Toxoplasma* to disseminate among cells and tissues like *C. trachomatis*. Nonetheless, the high abundance of PV membranous extensions in virulent TgNCR1-deficient parasites leads to the assumption that they could contribute to the pathogenicity.

In conclusion, the phenotype of NPC1 deficiency in *Toxoplasma* mimics in part the defects that have been observed in mammalian cells and yeast lacking functional NPC1 proteins, regarding the storage of multiple sphingolipid species. The accumulation of cholesteryl esters in parasite mutants, as observed in hepatocytes but not in other mammalian cells [59], is rather intriguing but may reflect the unusual properties of cholesterol metabolism in the parasite. Our results suggest that TgNCR1 may be an important contributor to lipid homeostasis in *T. gondii*. How precisely this protein exerts its action, e.g. as a detector of lipid levels in membranes, lipid translocator or negative regulator of lipid production, remains to be elucidated. One challenge for the future is to clarify the source of sphingolipids for the parasite, and the regulation and coordination of sphingolipid production with regards to parasite growth and virulence. Lipid molecules play an integrated role in human disease, and when one of them is misregulated, pathology frequently ensues. The control of levels of many lipids is obviously lost in the ΔNCR1 strain, and yet these parasites can overcome these lipidic perturbations and remain infective. On the one hand, these parasites adeptly respond to NCR1 loss by producing lipid bodies for the storage of excess lipids. On the other hand, TgNCR1-deficient *Toxoplasma* seems proficient in using lipids to build new membranes for organelle biogenesis. The ΔNCR1 strain provides an attractive model for studying cell division in Apicomplexa. In pursuit of a deeper understanding of the coordination of this process, our mutant parasites allow observations of the dynamics of the IMC and collection of ultrastructural details about organelle partitioning between descendants.

## Materials and Methods

### Ethics statement

All animal procedures were approved by the Institutional Animal Care and Use Committee of the Johns Hopkins University following the National Institutes of Health guidelines for animal housing and care.

### Chemicals and antibodies

All chemicals were obtained from Sigma (St Louis, MO) or Fisher (Waltham, MA) unless indicated otherwise. Solvents and standards for chromatography were of the highest analytical grade. Silica gel 60 thin-layer chromatography (TLC) plates (Merck KgAG, Darmstadt, Germany) were purchased through EM Science (Gibbstown, NJ). Radiolabeled reagent [5,6-^3^H] uracil (40 Ci/mmol) was purchased from Amersham Corp. (Arlington Heights, IL, USA). LysoTracker Red DND-99 was obtained from Invitrogen (Carlbad, CA). Ganglioside, sphingomyelin and ceramide standards were purchased from Avanti Polar Lipids (Alabaster, AL). Primary antibodies used in this study were: commercial mouse antibodies against HA (Roche Applied Science, Indianapolis, IN), *myc* (Cell Signaling Technology, Boston, MA), EEA1 (Abcam, Cambridge, MA) and LAMP1 (BD Biosciences, Palo Alto, CA), commercial rabbit antibody against *myc* (Santa Cruz Biotechnology, Santa Cruz, CA), rabbit polyclonal antibody against TgGRA7 [3] and mouse monoclonal antibody against TgGRA3 generously provided by J.F. Dubremetz (University of Montpellier).

### Mammalian cell lines, culture conditions and parasite propagation

Mammalian cell lines used included primary human foreskin fibroblasts (HFF), Chinese hamster ovary cells (CHO cells) and the somatic 2-2 mutant CHO cells with defective NPC1 kindly provided by L. Liscum (Tufts University) [29]. All of these cells were grown as monolayers and cultivated in α-minimum essential medium (MEM) supplemented with 10% fetal bovine serum (FBS), 2 mM glutamine and penicillin/streptomycin (100 units/ml per 100 µg/ml) as described [2]. The tachyzoite RH strain of *Toxoplasma gondii* and modified strains were propagated in vitro by serial passage in HFF [68]. To evaluate parasite viability, the measurement of [^3^H]uracil incorporated into the parasites was determined as described [69].

### Sequence analysis

Nucleotide and amino acid sequences were searched in the *T. gondii* database (www.toxodb.org) and the NCBI database using the BLAST algorithm [70]. Multiple sequence alignments were created using ClustalW, and the resulting similarities were then visualized by subjecting the alignment to BioEdit. Percent identity and similarity were calculated using standard tool for sequence analysis from NCBI (ncbi.nlm.nih.gov).

### Cloning of full-length cDNA encoding TgNCR1

Based on the blast search results of the ToxoDB, the ORF of the *Toxoplasma* homolog for NPC1 (TgNCR1: TGME49_090870) was amplified from a *T. gondii* sporozoite cDNA library generously provided by M.W. White (Montana State University) by using the primers F-TgNCR1-P1 and R-TgNCR1-P2 (see Table S2 for the sequences of primers used in this study). Amplified fragments (∼3.5-kb) were subcloned into pCR2.1 using the TOPO-TA cloning protocol (Invitrogen) and the insertion was confirmed by enzymatic digestion with EcoRI and sequencing. The cDNA sequence of TgNCR1 has been deposited in GenBank under the accession number JF836804.

### Plasmid constructs for expression in mammalian cells

Three plasmids were engineered for functional and localization studies of TgNCR1. The pcDNA3.1 vector allowing the expression of proteins with a *myc* tag at the C-terminus driven by a CMV promoter was used as a backbone. For the construction of the plasmid pTgNCR1-*myc*, the 5′ and 3′ ends of the *TgNCR1* sequence were PCR-modified using primers TgNCR1-P1 and TgNCR1-P2. The resulting PCR fragment was digested with EcoRI and HindIII before ligation into the same restriction sites in the pcDNA3.1 vector. This construct allows the expression of TgNCR1 in mammalian cells with a C-terminal *myc* tag. We also expressed a hybrid construct in which the C-terminal 78 residues of TgNCR1 were replaced with amino acids 1214 to1278 of human NPC1 by fusion PCR to generate the plasmid pTgNCR1-hNPC1. After RNA extraction from HFF, the coding sequence corresponding to the amino acids 1214-1278 of human NPC1 was amplified by RT-PCR (SuperScript One-Step RT-PCR with Platinum Taq, Invitrogen) using primers F-hNPC1_1214-1278_ and R-hNPC1_1214-1278_. The construct TgNCR1_Δ1101-1178_ was created by PCR amplification using F-*myc*-TgNCR1 _1-1101_ and R-TgNCR1_1-1101_. The two fragments were fused by PCR and amplified using the primers F-*myc*-TgNCR1_1-1101_ and R-hNCR1_1214-1278_. The fusion product was then ligated into pcDNA3.1 vector through EcoRI and HindIII to generate pTgNCR1-hNPC1. In this plasmid, a *myc* tag is introduced at the N-terminus of the resulting protein while the expression of the C-terminal *myc* tag originally present in vector pcDNA3.1. was blocked by introducing a stop codon in the primer R-hNPC1. Using the same cloning strategy, a plasmid control combining residues 938-1100 of TgNCR1 with residues 1214-1278 of human NPC1 named tr.(truncated) TgNCR1-hNPC1, was constructed with a N-terminal *myc* tag. Primers used for the fusion PCR were F-*myc*-TgNCR1_938-1100_, R-*myc*-TgNCR1_938-1100_, F-hNPC1_1214-1278_ and R-hNPC1_1214-1278_. For expression analysis in mammalian cells, cells were transfected with the different plasmids using lipofectamine method (Invitrogen).

### Plasmid construction for expression in *Toxoplasma*

To generate a strain of *T. gondii* stably expressing TgNCR1 wild-type or mutants with a HA tag at the N-terminus, we used the backbone of the parasite expression vector psagCATsag_Tub2358YFP obtained from the Roos laboratory (University of Pennsylvania) to construct pHA-TgNCR1. The TgNCR1 sequence was modified by PCR using the primers F-HA-TgNCR1 that contains the coding sequence of an HA tag for expression at the N-terminus and R-YFP-TgNCR1 that includes a stop codon to block the translation of the downstream YFP. Same approach was used for the construction of four plasmids harboring punctual mutations in the TgNCR1 sequence. The mutations of the targeted residues were carried out by fusion PCR. Two flanking primers were F- HA-TgNCR1 and R-YFP-TgNCR1, and the internal primers used were: F-TgNCR1_D571N_ and R-TgNCR1_D571N_ to generate pHA-TgNCR1_D571N_; F-TgNCR1_P913A_ and R-TgNCR1_P913A_ for pHA-TgNCR1_P913A_; F-TgNCR1_I957T_ and R-TgNCR1_I957T_ for pHA-TgNCR1_I957T_; F-TgNCR1_L1100V_ and R-TgNCR1_L1100V_ for pHA-TgNCR1_L1100V_. To create a strain of T. gondii transiently expressing TgNCR1 with a HA tag at the N-terminus under the NTPase promotor, we used the backbone of the parasite expression vector pRab5-HA containing the nucleoside triphosphatase III (NTPase III) promoter obtained from the Joiner laboratory (Yale University). The coding sequence of TgNCR1 was PCR-amplified using the primers F-haNTP and R-haNTP. The resulting PCR fragment was digested with SpeI and PacI, and directly ligated into the NheI and PacI sites of the pRab5-HA vector. In this construct, TgNCR1 was cloned in frame with the upstream HA tag coding sequence in the vector. To generate a strain of *T. gondii* transiently expressing TgNCR1 with a HA tag at the C-terminus under the tubulin promotor, the TgNCR1 sequence was modified by PCR using the primers F-NCRHA and R-NCRHA. The resulting PCR was digested by BamHI and AvrII and ligated into the BglII and AvrII sites of the sagCATsag_Tub2358YFP vector. The reverse primer contained the coding sequence of HA tag followed by a stop codon to block the translation of the downstream YFP tag. For transient transfection in *T. gondii*, expression plasmids containing TgNCR1coding sequences, wild-type or mutants, were transformed in *E. coli* DH-5α and isolated using Qiagen Plasmid Purification Kits. Parasites were transfected by electroporation as described [6].

### Genetic disruption of TgNCR1 gene

To create the ΔNCR1 strain, a fusion PCR knockout construct was generated, consisting of 3′ and 5′*NCR1* genomic flanks fused on either site of the hypoxanthine-xanthine-guanine phosphoribosyltransferase (HXGPRT) selectable marker cassette (Figure S9). Genomic flanking sequences of the *TgNCR1* gene were obtained from the *Toxoplasma* database (www.toxodb.org; version 4.3). Primers were designed to amplify ∼550-bp of each of the two flanking regions from the genomic DNA of the RH strain of *Toxoplasma*. Two flanks were amplified to overlap on one end with the HXGPRT selectable marker cassette, and similarly, the HXGPRT marker cassette was amplified from pminiHXGPRT plasmid to incorporate the TgNCR1 sequence on the other end. The three individual fragments corresponding to 5′ flank, 3′ flank and HXGPRT were obtained by PCR reactions using primers KOPCR-1 and KOPCR-4, KOPCR-5 and KOPCR-2, KOPCR-3 and KOPCR-6, respectively. The three PCR fragments were then used as template to generate the fusion construct using primers KOPCR-1 and KOPCR-2 as shown in Figure 5. Deletion of *TgNCR1* from the parasite genome and HXGPRT arrangement were verified by PCR and Southern blot analyses. For PCR confirmation, genomic DNA was isolated from the colonies obtained through selection and used as template for PCR. Replacement of *TgNCR1* by *HXGPRT* was monitored using a primer outside the 5′ genomic flanking region (P1) and a primer within the selectable marker HXGPRT (P2). The primer pairs P3-P4 and P5-P6 were used to assert the absence of *TgNCR1*/the presence of a single copy of *HXGPRT* in the ΔNCR1 strain and the presence of *TgNCR1*/the absence of *HXGPRT* in the ΔKu80ΔHXGPRT (ΔK80) parental strain [71], respectively. For Southern blotting, the primers F-NPC-sb-1 and R-NPC-sb-1 were used in the PCR to generate a ∼0.9-kb probe specific for the *HXGPRT* gene. Genomic DNA samples (80 µg) were digested with AgeI and electrophoresed through 0.7% agarose gels. The DNA was then transferred onto a Hybond N^+^ membrane (Biotech) and hybridized randomly with the ^32^P-labeled DNA probe. Blots were then washed at appropriate stringency and visualized by autoradiography.

### Selection of parasite stable lines

For the generation of the ΔNCR1 strain, fusion PCR products (15–20 µg) containing 5′ UTR of *TgNCR1* gene, HXGPRT cassette and 3′ UTR of *TgNCR1* gene were electroporated into the parasite ΔKu80 strain [71] as described [6]. After overnight growth, transformants were placed under 25 µg/ml of mycophenolic acid and 50 µg/ml of xanthine. Transformant pools were tested by PCR to validate the deletion of *NCR1* in parasites, and TgNCR1 knockout parasites were further selected by limiting dilution under drug selection. Individual knockout clones were then plated in 96-well plates and cultivate to ensure clonality.

### Fusion PCR construct for complementation

To create the construct for the complementation of the ΔNCR1 strain, the same fusion PCR approach shown in Figure S9 was used. Two fragments, 5′ and 3′ flanking sequences of the *NCR1* gene, were amplified using the primers KOPCR-1 and Ncomp-4, Ncomp-5 and KOPCR-2, respectively, to overlap on one end with the TgNCR1 coding sequence. The third fragment, TgNCR1 coding sequence, was amplified using primers Ncomp-3 and Ncomp-6. The three PCR fragments were then used as template to generate the fusion construct using the primers KOPCR-1 and KOPCR-2.

### Negative selection of HXGPRT with 2-hydroxy-6-mercaptopurine

For the complementation of the ΔNCR1 strain, 25 µg of the fusion PCR product was electroporated into the ΔNCR1 strain. Twenty four hours post-transfection, the culture media of transformants was replaced with DMEM containing HEPES and 1% dialyzed FBS, and the tranformants were placed under the selection of 0.85 µg/ml of 2-hydroxy-6-mercaptopurine. Drug resistant transformant pools were tested by PCR to validate the replacement of *HXGPRT* with *NCR1* in parasites, and NCR1-complemented parasites were further selected by limiting dilution in 96-well plates. Clones were then tested using drug selection to ensure that they grow in 2-hydroxy-6-mercaptopurine but no longer in mycophenolic acid and xanthine. After the selection, the 5′ integration of TgNCR1 coding sequence was confirmed by PCR using the pair of primers P1-P7, and the 3′integration was verified by PCR using the pair of primers P8-P9.

### Recombinant peptide expression in *E. coli* and affinity purification

To generate antibodies against TgNCR1, we engineered a plasmid to produce a recombinant peptide corresponding to the sequence of TgNCR1_162-501_. The coding sequence corresponding to TgNCR1_162-501_ was PCR-amplified using the primers F- TgNCR1_162-501_ and R-TgNCR1_162-501_ and directly cloned into the BamHI and HindIII sites of the pQE-30 vector (Qiagen, Hilden, Germany) to generate N-terminal 6-His tagged fusion protein. The recombinant peptide expressed in *E. coli* M15 strain were purified under denatured condition on Ni^2+^-NTA resin according to the Qiagen protocol. After purification, the peptide was refolded by diluting the sample 10 times in the refolding buffer (50 mM Tris/HCl pH 7.5, 1 mM EDTA, 1 M L-arginine, 1 mM reduced form of glutathione, 0.8 mM of oxidized form of glutathione) at 4°C overnight before concentration and dialysis against phosphate-buffered saline (PBS). Some batches of antibodies against TgNCR1_162-501_ were further preadsorbed on a fibroblast lysate (overnight, 4°C) to diminish unspecific cross-reactions between the primary antibody and host cell epitopes.

### Immunoblot analysis of parasites stably expressing HA-TgNCR1

For immunodetection, transgenic parasites were lysed in the M-PER mammalian protein extraction reagent (Pierce Biotechnology, Rockford, IL). The cell extracts were suspended in SDS gel-loading buffer (50 mM Tris–HCl (pH 6.8), 50 mM 2-mercaptoethanol, 2% SDS, 0.1% bromophenol blue, 10% glycerol) and lysed by boiling in a water bath. The samples were subjected to SDS-PAGE, and the proteins were then electrophoretically transferred to a membrane (Immobilon Transfer Membranes, Millipore, Bedford, MA). The membrane was immersed in blocking buffer (PBS containing 3% skim milk) for 60 min, and then incubated with anti-HA antibody (1∶5000) in the blocking buffer for 60 min. Unbound antibody was removed by washing the membrane six times with blocking buffer. Next, the membrane was incubated with horseradish peroxidase-conjugated goat anti-mouse IgG antibody (Amersham Pharmacia Biotech; dilution, 1∶1000) in blocking buffer for an additional hour, before detection by chemiluminescence.

### Cell cycle analysis

Vero cells were infected with either the ΔNCR1 or parental strain at the M.O.I of 10 and washed every 2 h p.i. to synchronize the infection. Fifteen hours p.i., infected cells were exposed to the calcium ionophore A23187 at 4 µM for 10 min to induce parasites egress. Parasites in the supernatant were collected by aspiration, washed by centrifugation and fixed in ice-cold ethanol (70%), followed by incubation with propidium iodide (PI) solution (0.01 mg/ml PI; 0.01 mg/ml DNAse-free RNAse A; 0.1% Triton X-100; 1 mg/ml sodium citrate) at 4°C overnight. Uninfected VERO cells as controls were treated similarly to infected cells. Flow cytometry analysis was performed on a FACScan flow cytometer (Becton Dickinson, Mountain View, CA). Cell cycle distribution pattern was assessed using FlowJo software (Tree Star, Inc.); G1, G2 and S peaks were defined using the Dean-Jet-Fox model.

### Mice and in vivo virulence assays

5 week-old female BALB/C mice were purchased from The Jackson Laboratory (Bar Harbor, ME). Groups of six mice were infected intraperitoneally with 5×10^4^ tachyzoites of ΔNCR1 or ΔKu80ΔHXGPRT parasites. The survival of mice after infection was monitored daily. Results of three independent experiments are presented.

### Measurement of lipids

#### Thin layer chromatography (TLC)

Total lipids from mammalian cells were extracted in chloroform/methanol (v/v, 2∶1), subsequently separated by TLC on silica plates using hexane:diethyl ether:acetic acid (v/v/v, 90∶10∶1) for free cholesterol and cholesteryl esters, or using chloroform:methanol:0.2% CaCl_2_ (v/v/v/, 60∶35∶8) for gangliosides, and visualized as described [31].

#### Mass spectrometry

Total lipids from the ΔNCR1 and parental strains were extracted using a modified Bligh and Dyer procedure as described [72]. Purified standards of lipids were added directly to homogenates. Mass spectrometry analyses of species of cholesterol, cholesteryl esters, triglycerides, sphingolipids and phospholipids were performed using a Sciex API 3000 triple stage quadruple tandem mass spectrometer (ESI/MS/MS) from Sciex Inc. (Thornhill, Ontario, Canada), using methods similar to those described in previous studies [72], [73]. Peaks were evaluated with non-parametric one-way ANOVA (Kruskal-Wallis test, with a threshold of *P*-value <0.05 considered as statistically significant.

### Light and electron microscopy studies

Light and epifluorescence microscopy were performed on infected cells seeded on sterile coverslips in 24-well culture dishes. IFA on parasites or mammalian cells were performed as previously described [56] using primary antibodies against GRA3 (1∶100), anti-GRA7 (1∶100), *myc* (1∶100), HA (1∶1000), EEA1 (1∶100), LAMP1 (1∶100), and secondary antibodies (Invitrogen): anti-mouse and anti-rabbit IgG antibodies conjugated to either Alexa 488 or Alexa 594 diluted at 1∶2000. For detection of cholesterol or lipid bodies by fluorescence microscopy, intravacuolar parasites were fixed in paraformaldehyde, and treated as described [2], [56]. Slides were observed using a Nikon Eclipse E800 microscope equipped with a Spot RT CCD Camera and processed using Image-Pro-Plus software (Media Cybernetics, Silver Spring, MD) before assembly using Adobe Photoshop (Adobe Systems, Mountain View, CA). For analysis of PV membranous extensions, HFF were infected with either the ΔNCR1 or ΔHXGPRT stain for 36-h. After fixation and immunostaining for GRA7 and GRA3, parasites were imaged with a fluorescent microscope (Nikon 90i) using a Plan-Apochromat 100x/1.4 NA. Serial optical z-sections were acquired with a Hammatsu camera and Volocity software (Improvision, Waltham, MA). Using the Volocity software, images were processed by iterative restoration (deconvolution algorithm) and the length and diameter of the PV membranous extensions, as detected by GRA3 staining, were measured. To quantify the levels of filipin accumulation, images were collected using sequential scanning, processed and merged using Volocity software. For ultrastructural observation of the ΔNCR1 strain by thin-section transmission electron microscopy (EM), infected cells were fixed in 2.5% glutaraldehyde (Electron Microscopy Sciences, Hatfield, PA) and processed as described [56]. Ultrathin sections of infected cells were stained before examination with a Philips CM120 EM (Eindhoven, the Netherlands) under 80 kV. For immunoelectron microscopy, *Toxoplasma*-infected cells were fixed in 4% paraformaldehyde (Electron Microscopy Sciences) and processed as previously described [38]. The sections were immunolabeled with anti-HA antibodies (1∶200 in PBS/1% fish skin gelatin), then with mouse anti-mouse IgG antibodies, followed directly by 10 nm protein A-gold particles (Department of Cell Biology, Medical School, Utrecht University, the Netherlands) before microscopic examination.

### Protein determination

Protein content was determined using the bicinchoninic acid assay [74] with serum bovine albumin (BSA) as a standard.

## Supporting Information

Figure S1 Predicted ORF of TgME49_090870 from *T. gondii.* The sterol-sensing-like domain is underlined in red. Potential transmembrane segments (according to the transmembrane folding program:http://liao.cis.udel.edu/website/servers/TMMOD/scripts/frame.php?p=description) are shown in yellow while putative N-glycosylation sites (according to the NetNGLyc 1.0 server) are in blue.
(PDF)

Figure S2 Phylogeny of NPC1-related proteins in the indicated organisms. The EuPath database version 2.10 was searched using the blastp tool with the protein sequence of TGME49_090870 (full-length) to identify NPC1-related proteins among the eukaryotic pathogens of the genera *Cryptosporidium*, *Giardia*, *Leishmania*, *Neospora*, *Plasmodium*, *Toxoplasma*, *Trichomonas* and *Trypanosoma*. Many Apicomplexa have one or two NPC1-like proteins. Among other protozoa, Entamoeba species have a NPC1-related protein, which shows close phylogeny to yeast, human and helminth NPC1 homologs. No NPC1-like gene was present in Flagellates (e.g., *Trypanosoma*, *Leishmania* and *Giardia*). The resulting full-length sequences were aligned using ClustalW (scoring matrix = Blosum; gap open penalty = 10; end gap penalty = 10; gap extension penalty = 0.5; separation gap penalty = 0.05). We have calculated an unrooted phylogenic tree according to the program Jalview 2.6.1 [22] by neighbor joining using percent identity.
(PDF)

Figure S3 Predicted transmembrane regions within the SSD of the indicated proteins. Multiple sequence alignment of the predicted SSD of TGME49_090870 with the human sequences of NPC1 (GI:38649260), Patched (GI:1381236) and SCAP (GI:66932902) using the CLUSTALW program. The five successive transmembrane domains are indicated in color.
(PDF)

Figure S4 Characterization of the 2-2 mutant CHO cell line. **A**. Fluorescence microscopy of the cells labeled with the filipin dye showing bright punctate structures resulting from the accumulation of cholesterol in endo-lysosomes. **B**. EM at high magnification of these cells showing cholesterol deposits as electron-dense materials in the overloaded lysosomes.
(PDF)

Figure S5 Cholesteryl ester (CE) levels in the mammalian NPC1-mutant cells expressing TgNCR1-hNPC1. Quantitative TLC analysis of neutral fraction of cellular lipid extracts from CHO wild-type cells or mutants transfected with either the vector only or TgNCR1-hNPC1. No significant differences in CE levels were detected between the 3 conditions (n = 3 independent assays).
(PDF)

Figure S6 Coomassie blue-stained gel showing the 38-kDa recombinant peptide from TgNCR1 after purification from *E. coli* extracts. The peptide was used to produce antibodies against TgNCR1.
(PDF)

Figure S7 Immunolocalization of TgNCR1 in wild-type *T. gondii.* IFA of intracellular parasites using anti-TgNCR1_162-501_ antibodies (dilution 1/5) showing a peripheral fluorescence signal on the parasites forming small or large PV. Arrows in red pinpoint stained structures that are suggestive for the inner membrane complex, which is located beneath the plasma membrane.
(PDF)

Figure S8 Localization of TgNCR1 in parasites transiently expressing either HA-TgNCR1or TgNCR1-HA. Immunofluorescence assays using anti-HA antibodies on parasites transiently transfected with a plasmid containing *HA-TgNCR1* under the NTPase promoter (A) or *HA-TgNCR1-HA* under the tubulin promoter (B) showing IMC labeling 16 h to 24 h p.i.
(PDF)

Figure S9 Schematic showing the *NCR1* knockout strategy. Double homologous recombination results in the replacement of *NCR1* with the selectable marker *HXGPRT*. *NCR1* 5′ and 3′ genomic flanking regions were amplified with sequences overlapping the dhfr-HXGPRT-dhfr selectable marker while the selectable marker was amplified to contain overlaps with the *NCR1* genomic flanking regions. Using fusion PCR, a ΔNCR1 product was created containing the genomic flanking regions of *NCR1* on either side of the selectable marker. Double homologous recombination was used to replace *NCR1* with *HXGPRT*.
(PDF)

Figure S10 Transmission EM of the ΔNCR1 strain infecting HFF for 48 h. Knockout parasites formed large rosettes within their PV similarly to the parental strain. Note the association of host mitochondria (hm) with the PV membrane and the retention of residual bodies (RB). Arrowheads show IMC profiles. Bar is 2 µm. The inset shows a phase-contrast image by light microscopy of the ΔNCR1 strain.
(PDF)

Figure S11 Complementation of the ΔNPC1 strain with *NCR1*from *Toxoplasma.* Top: schematic showing the two primer sets used to verify the insertion of NCR1 into the ΔNCR1 strain. The 5′ integration of *NCR1* coding sequence was confirmed by PCR analysis using P1 and P7, and the 3′ integration by PCR using P8 and P9.
(PDF)

Figure S12 Ultrastructural observations of TgNCR1-deficient parasites. Transmission EM of TgNCR1-deficient parasites infecting HFF for 24 h. A. Replicating parasites were frequently observed for the ΔNCR1 strain, indicative of synchronous division. Images from panel a to g illustrate progressive views of parasite endodyogeny, presenting similarities with wild-type parasites. Arrows pinpoint IMC scaffolds. a, apicoplast; Go, Golgi; m, mitochondrion; mi, micronemes; n, nucleus; r, rhoptries. Bars are 1 µm.
(PDF)

Figure S13 Demonstration of tubular extensions from the PV of wild-type parasites (RH strain). Immunofluorescence assays using anti-GRA7 antibodies on *Toxoplasma*-infected cells after 24 h. Note the presence of at least three PV membrane extensions into the host cytosol.
(PDF)

Table S1 Analysis of phospholipid composition in the ΔNCR1 and parental strains by mass spectrometry. Quantitative analysis of MRM spectra from the ΔNCR1 and parental strains showing levels of various species of phospholipids. Statistically significant differences in the amounts of few species of phosphatidylethanolamine and phosphatidylserine were detected in TgNCR1-deficient parasites. Data are cps means ± SD from 3 independent populations from each strain.
(PDF)

Table S2 Primers used in this study.
(PDF)
