# Supplementary material for: Deficiency of a Niemann-Pick, Type C1-related Protein in Toxoplasma Is Associated with Multiple Lipidoses and Increased Pathogenicity
Source: PLoS Pathog. 2011 Dec 8;7(12):e1002410. doi: 10.1371/journal.ppat.1002410 (PMC3234224; doi:10.1371/journal.ppat.1002410)
Supplement: Figure S1 — Predicted ORF of TgME49_090870 from T. gondii. The sterol-sensing-like domain is underlined in red. Potential transmembrane segments (according to the transmembrane folding program:http://liao.cis.udel.edu/website/servers/TMMOD/scripts/frame.php?p=description) are shown in yellow while putative N-glycosylation sites (according to the NetNGLyc 1.0 server) are in blue. (PDF) [file ppat.1002410.s001.pdf]

MEKNCNSVAGHAGTLVKASSRQADDKAGSRVPPGGHLLQT**N**KTESQLLPCASPSDPVHWT 60

NEIVPVVADTSAPAEAAPDQTVSDGEQLTRAPDETYYQRSESLAEPPAENELPAGRSRRR 120

RSRWCLGCGCFEQVKTVVLRMLMTGFEKYAGVVYDHPWLFIMVSLLATAGMSVGIFLRTP 180

ESDVYTLYSLSGSPSQVTKEHLLDVLPPDRLLFVLLTGTSNLVTRETVTRIDSLLQGIES 240

ITLRRDSVTTDEFNHRLVSHDRSPFPETITFQDICAKDGSGKCQVQSILDLYPSSSAWGV 300

MPIASASWPVVTNPVTHKVSRLDAILGKITTSVRLAEQGSGRPALTVVEEAEAMLMRIDL 360

RGETIWKPYTAAFEKLVLDYVLGQDFGPDISVTAKAERSSYDELKRVSTLDVVEWLRLCA 420

AVLVVFLYTSVVNSSKTHRTKLVPSAMGALASLLGYLGGAGLVYLCGVRHTTPAEATPFL 480

AIGIGVDDLFVIINAYSLTYLHPNPKERVVDAIRDAGLSITITTLTNVITFIIGALSPYY 540

SISMFCIITAGALTWGYVLCLTFFLAGLSLDARREARKEPLSYSLFWRFMPRCCRKSSYE 600

PQLSPPLPLTAAASGLEEMR**N**ESEEIVTADQTPPTHGGDLLTTYQLAALMVLYKKHTCQS 660

RSSQPARRLFDRRDGTGTEETMSTPVPEDSERDRTNQKSCGTQVSTGIVDVRETGQDAAS 720

QRRLSSHIRDMTTQESIMLLKNFEKEMEQNPEKLLKLYHPEPLGNPGRGSRRFFRDYYGR 780

FLGNTFVKATVLVIFAAVTALAIYGATTLKFGLSLKNITPQASYLRDFYSLHEDLFPSYG 840

DEVTVFFAENDRWEDREVQMRYLQMVKELSEQEWAVVVTDGMSLFLQHAMPSLHSGNRKE 900

FLALLKTWLEGDPIGQ**N**FSTFFKFSFDNLIVWQFRYWMPHRD**N**TTTLYYWLKEGKDIVSA 960

GKPYFHGEVHTALAVIWESDPKILPFTLTNLSIALVCILAISLLLIPDLTSAIIVVLVVS 1020

LVDLWLFGFMALIDLPLSMISMVNLLISIGYSVDFTIHVAHTFTHCVGASRKDRMVETMI 1080

VMGAPVTHGMLSTLLSILALAGSPKYILEVFFKMMFMVIVFAYTAGMVLLPVVLTLLGPF 1140

HPHGKRESGKAIACDSSAQLIDMEPLHGTGKEEHGVGV 1178
