# Supplementary material for: Deficiency of a Niemann-Pick, Type C1-related Protein in Toxoplasma Is Associated with Multiple Lipidoses and Increased Pathogenicity
Source: PLoS Pathog. 2011 Dec 8;7(12):e1002410. doi: 10.1371/journal.ppat.1002410 (PMC3234224; doi:10.1371/journal.ppat.1002410)
Supplement: Figure S2 — Phylogeny of NPC1-related proteins in the indicated organisms. The EuPath database version 2.10 was searched using the blastp tool with the protein sequence of TGME49_090870 (full-length) to identify NPC1-related proteins among the eukaryotic pathogens of the genera Cryptosporidium, Giardia, Leishmania, Neospora, Plasmodium, Toxoplasma, Trichomonas and Trypanosoma. Many Apicomplexa have one or two NPC1-like proteins. Among other protozoa, Entamoeba species have a NPC1-related protein, which shows close phylogeny to yeast, human and helminth NPC1 homologs. No NPC1-like gene was present in Flagellates (e.g., Trypanosoma, Leishmania and Giardia). The resulting full-length sequences were aligned using ClustalW (scoring matrix = Blosum; gap open penalty = 10; end gap penalty = 10; gap extension penalty = 0.5; separation gap penalty = 0.05). We have calculated an unrooted phylogenic tree according to the program Jalview 2.6.1 [22] by neighbor joining using percent identity. (PDF) [file ppat.1002410.s002.pdf]

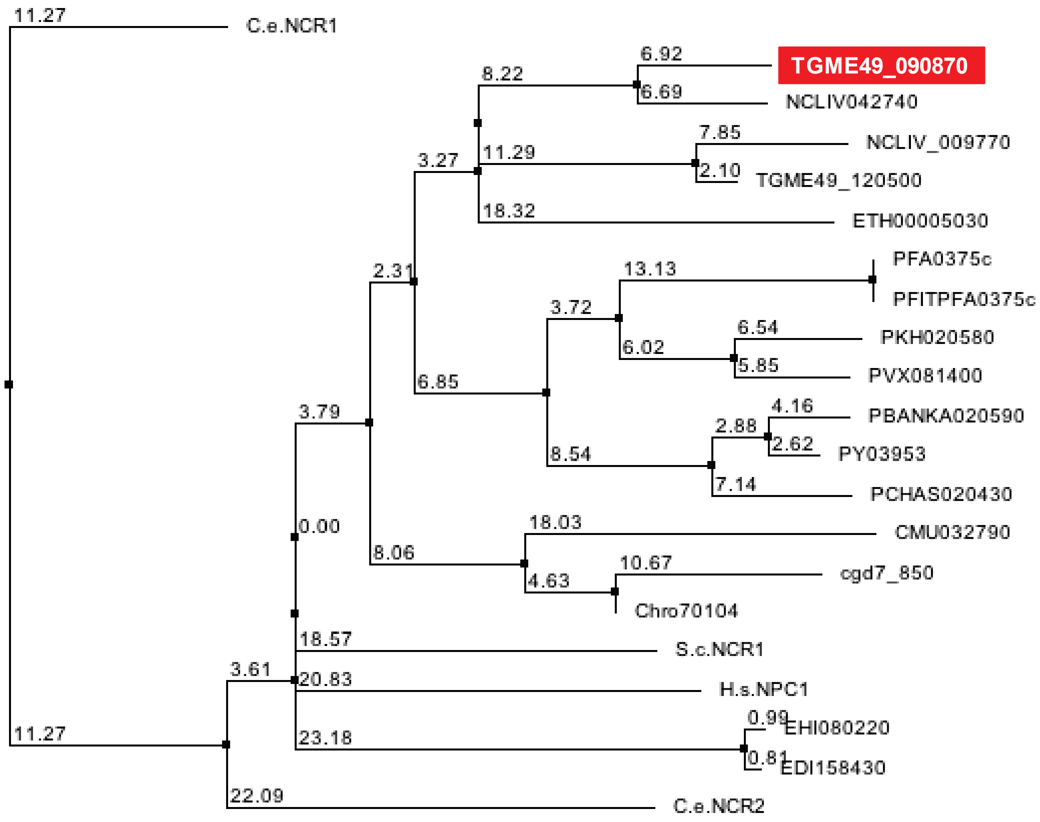

TGME49_090870 and TGME49_120500: ***Toxoplasma gondii*** **(str. ME49)**
NCLIV: ***Neospora caninum*** **(str. LIV)**
ETH: ***Eimeria tenella*** **(str. Hough)**
PFA0375c: ***Plasmodium falciparum*** **(str. 3D7)**
PFIT_PFA0375c: ***Plasmodium falciparum*** **(str. IT)**
PKH: ***Plasmodium knowlesi***
PVX: ***Plasmodium vivax*** **(str. SaI-1)**
PBANKA: ***Plasmodium berghei*** **(str. ANKA)**
PY: ***Plasmodium yoelii***
PCHAS: ***Plasmodium chabaudi***
CMU: ***Cryptosporidium muris***
cgd7: ***Cryptosporidium parvum***
Chro: ***Cryptosporidium hominis***
EHI: ***Entamoeba histolytica*** **(str. HM-1)**
EDI: ***Entamoeba dispar*** **(str. AW760)**
H.s.: ***Homo sapiens***
S.c.: ***Saccharomyces cerevisiae***
C.e.: ***Caenorhabditis elegans***
