# Supplementary material for: Deficiency of a Niemann-Pick, Type C1-related Protein in Toxoplasma Is Associated with Multiple Lipidoses and Increased Pathogenicity
Source: PLoS Pathog. 2011 Dec 8;7(12):e1002410. doi: 10.1371/journal.ppat.1002410 (PMC3234224; doi:10.1371/journal.ppat.1002410)
Supplement: Figure S3 — Predicted transmembrane regions within the SSD of the indicated proteins. Multiple sequence alignment of the predicted SSD of TGME49_090870 with the human sequences of NPC1 (GI:38649260), Patched (GI:1381236) and SCAP (GI:66932902) using the CLUSTALW program. The five successive transmembrane domains are indicated in color. (PDF) [file ppat.1002410.s003.pdf]

```
TGME49_090870  LDVVEWLRLCAAVLVVFLYTSVVNSSKTHRT------KLVPSAMGALASLLGYLGGAGLV  463
hNPC1          ESDSDVFTVVISYAIMFLYISLALGHIKSCRRLLVDSKVSLGIAGILIVLSSVACSLGVF  675
hPatched       FSDVSVIRVASGYLLMLAYACLTMLRWDCSK-----SQGAVGLAGVLLVALSVAAGLGLC  488
hSCAP          IGVAELIPLVTTYIILFAYIYFSTRKIDMVK-----SKWGLALAAVVTVLSSLLMSVGLC  334

TGME49_090870  YLCGVRHTTP-AEATPFLAIGIGVDDLFVIINAYSLTY--LHPNPKERVVDAIRDAGLSI  520
hNPC1          SYIGLPLTLIVIEVIPFLVLAVGVDNIFILVQAYQRDERLQGETLDQQLGRVLGEVAPSM  735
hPatched       SLIGISFNAATTQVLPFLALGVGVDDVFLLAHAFSETGQNKRIPFEDRTGECLKRTGASV  548
hSCAP          TLFGLTPTLNGGEIFPYLVVVIGLENVLVLTKSVVSTP--VDLEVKLRIAQGLSSESWSI  392

TGME49_090870  TITTLTNVITFIIGALSPYYSISMFCIITAGALTWGYVLCLTFFLAGLSLDARR------  574
hNPC1          FLSSFSETVAFFLGALSVMPAVHTFSLFAGLAVFIDFLLQITCFVSLLGLDIKRQE----  791
hPatched       ALTSISNVTAFFMAALIPIPALRAFSLQAAVVVVFNFAMVLLIFPAILSMDLYRREDRRL  608
hSCAP          MKNMATELGIILIGYFTLVPAIQEFCLFAVVGLVSDFFLQMLFFTTVLSIDIRR------  446
```
