# Supplementary figures and images for: Deficiency of a Niemann-Pick, Type C1-related Protein in Toxoplasma Is Associated with Multiple Lipidoses and Increased Pathogenicity
Source: PLoS Pathog. 2011 Dec 8;7(12):e1002410. doi: 10.1371/journal.ppat.1002410 (PMC3234224; doi:10.1371/journal.ppat.1002410)

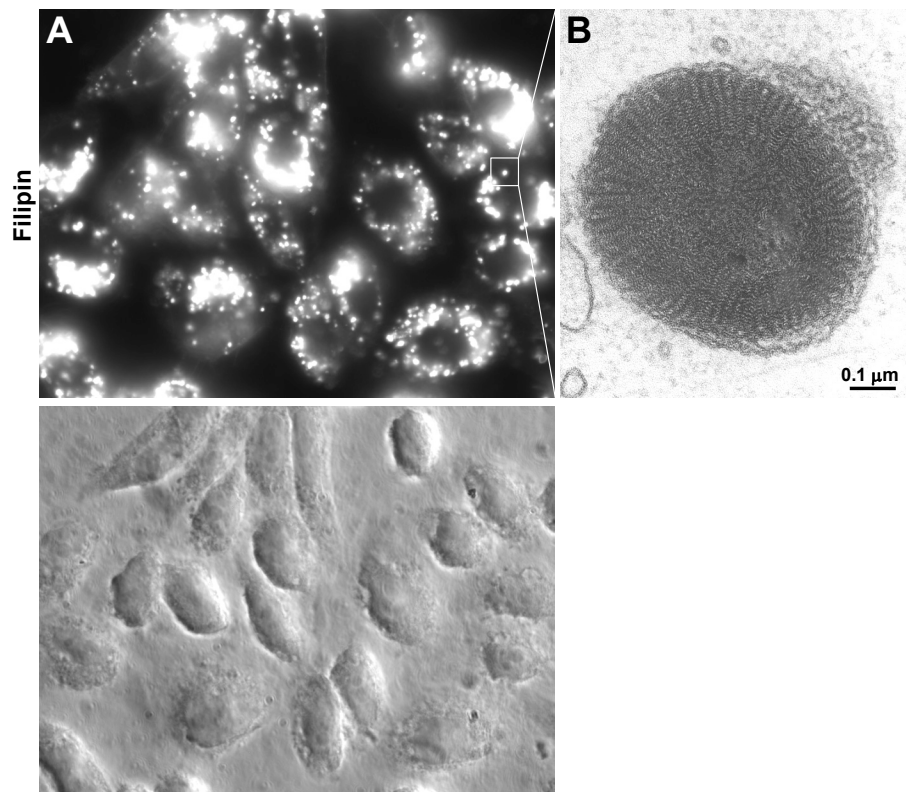

Supplement: Figure S4 — Characterization of the 2-2 mutant CHO cell line. A. Fluorescence microscopy of the cells labeled with the filipin dye showing bright punctate structures resulting from the accumulation of cholesterol in endo-lysosomes. B. EM at high magnification of these cells showing cholesterol deposits as electron-dense materials in the overloaded lysosomes. (PDF) [file ppat.1002410.s004.pdf]

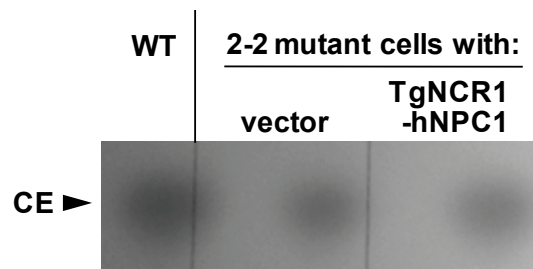

Intensity (A.U.)

Mean:

1.37

1.11

1.24

SD:

0.41

0.37

0.5

Supplement: Figure S5 — Cholesteryl ester (CE) levels in the mammalian NPC1-mutant cells expressing TgNCR1-hNPC1. Quantitative TLC analysis of neutral fraction of cellular lipid extracts from CHO wild-type cells or mutants transfected with either the vector only or TgNCR1-hNPC1. No significant differences in CE levels were detected between the 3 conditions (n = 3 independent assays). (PDF) [file ppat.1002410.s005.pdf]

MWt (kDa)

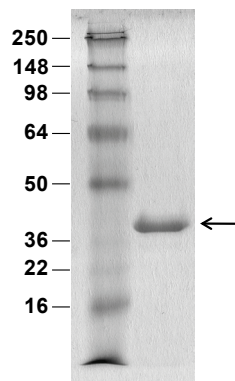

Supplement: Figure S6 — Coomassie blue-stained gel showing the 38-kDa recombinant peptide from TgNCR1 after purification from E. coli extracts. The peptide was used to produce antibodies against TgNCR1. (PDF) [file ppat.1002410.s006.pdf]

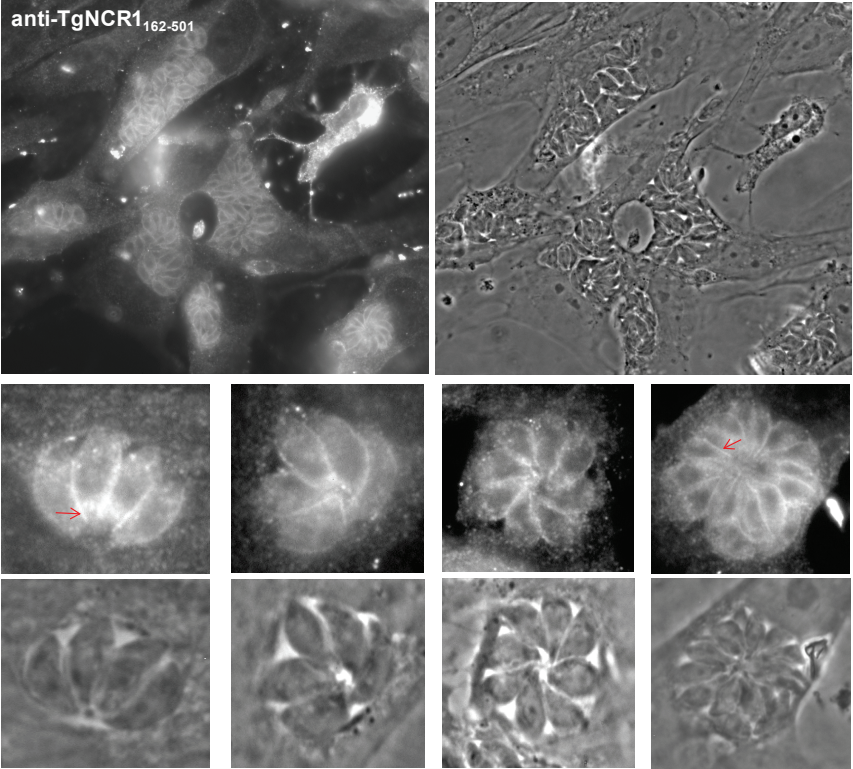

Supplement: Figure S7 — Immunolocalization of TgNCR1 in wild-type T. gondii. IFA of intracellular parasites using anti-TgNCR1162-501 antibodies (dilution 1/5) showing a peripheral fluorescence signal on the parasites forming small or large PV. Arrows in red pinpoint stained structures that are suggestive for the inner membrane complex, which is located beneath the plasma membrane. (PDF) [file ppat.1002410.s007.pdf]

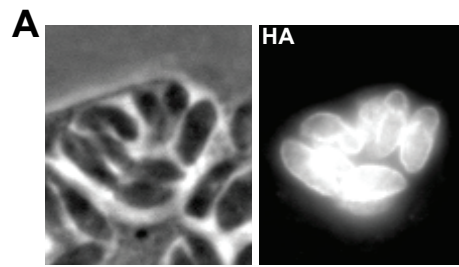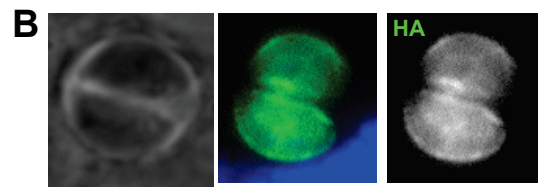

Supplement: Figure S8 — Localization of TgNCR1 in parasites transiently expressing either HA-TgNCR1or TgNCR1-HA. Immunofluorescence assays using anti-HA antibodies on parasites transiently transfected with a plasmid containing HA-TgNCR1 under the NTPase promoter (A) or HA-TgNCR1-HA under the tubulin promoter (B) showing IMC labeling 16 h to 24 h p.i. (PDF) [file ppat.1002410.s008.pdf]

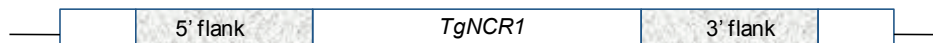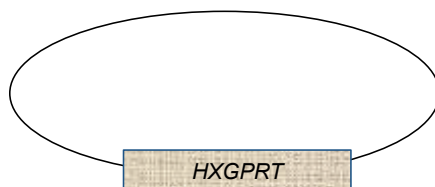

↓ PCR

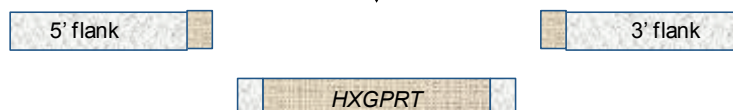

↓ Fusion PCR

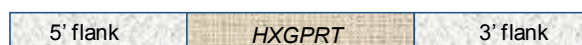

Supplement: Figure S9 — Schematic showing the NCR1 knockout strategy. Double homologous recombination results in the replacement of NCR1 with the selectable marker HXGPRT. NCR1 5′ and 3′ genomic flanking regions were amplified with sequences overlapping the dhfr-HXGPRT-dhfr selectable marker while the selectable marker was amplified to contain overlaps with the NCR1 genomic flanking regions. Using fusion PCR, a ΔNCR1 product was created containing the genomic flanking regions of NCR1 on either side of the selectable marker. Double homologous recombination was used to replace NCR1 with HXGPRT. (PDF) [file ppat.1002410.s009.pdf]

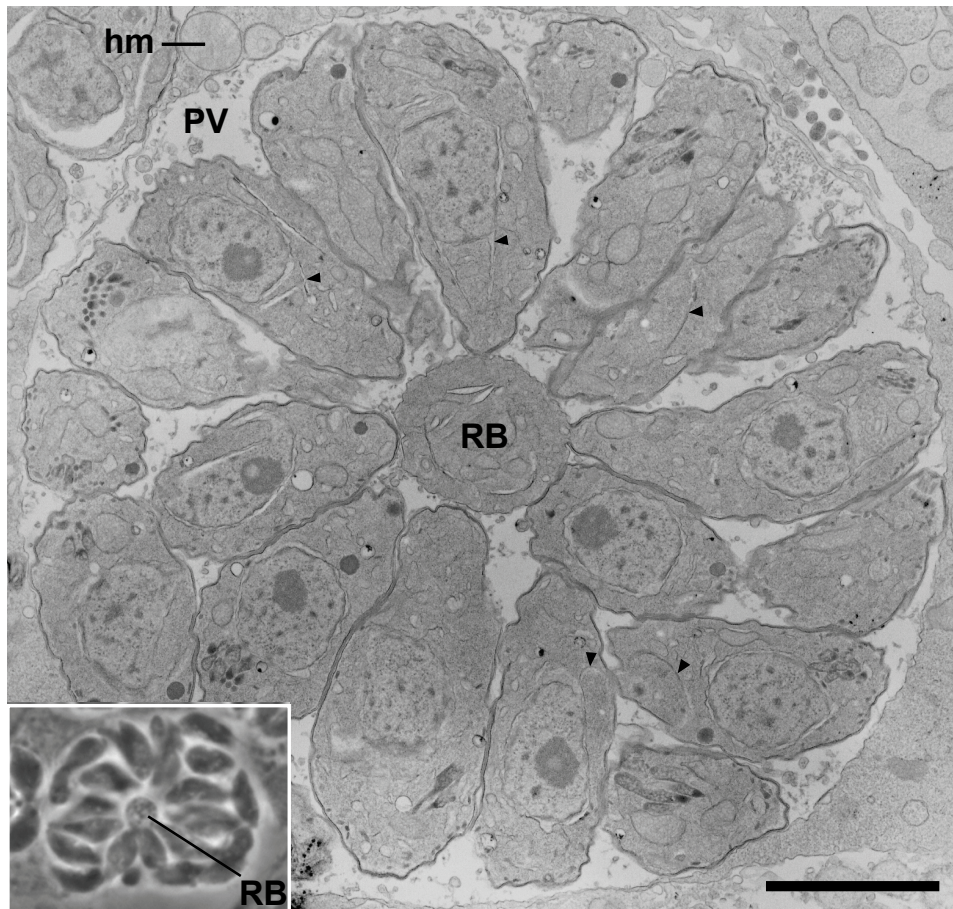

Supplement: Figure S10 — Transmission EM of the ΔNCR1 strain infecting HFF for 48 h. Knockout parasites formed large rosettes within their PV similarly to the parental strain. Note the association of host mitochondria (hm) with the PV membrane and the retention of residual bodies (RB). Arrowheads show IMC profiles. Bar is 2 µm. The inset shows a phase-contrast image by light microscopy of the ΔNCR1 strain. (PDF) [file ppat.1002410.s010.pdf]

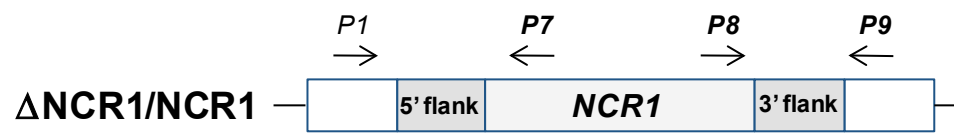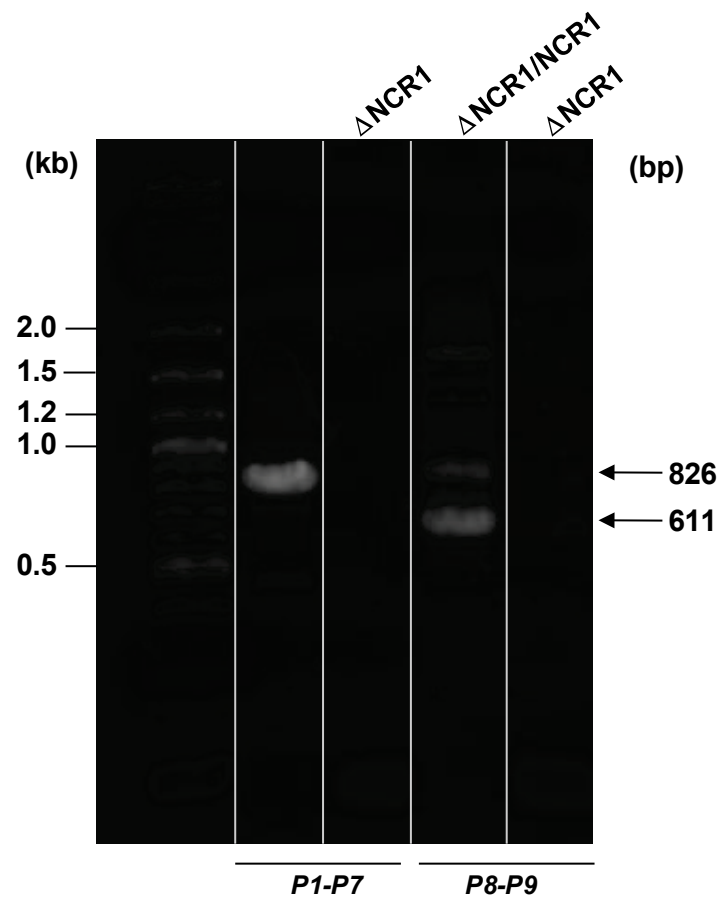

Supplement: Figure S11 — Complementation of the ΔNPC1 strain with NCR1from Toxoplasma. Top: schematic showing the two primer sets used to verify the insertion of NCR1 into the ΔNCR1 strain. The 5′ integration of NCR1 coding sequence was confirmed by PCR analysis using P1 and P7, and the 3′ integration by PCR using P8 and P9. (PDF) [file ppat.1002410.s011.pdf]

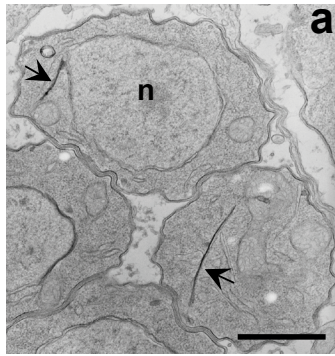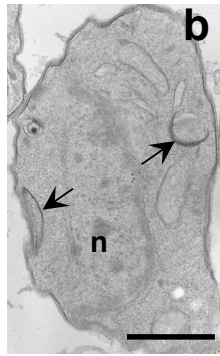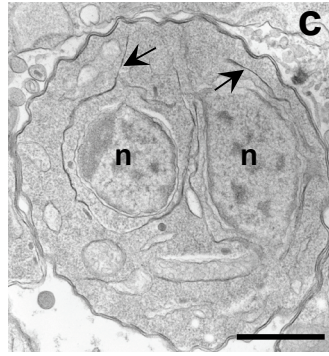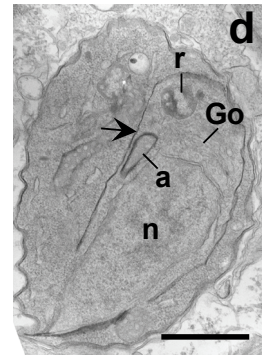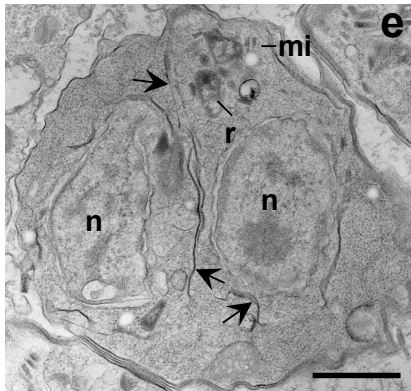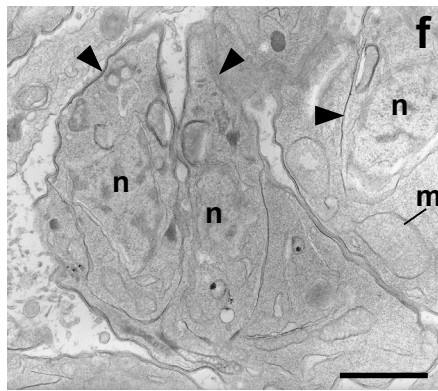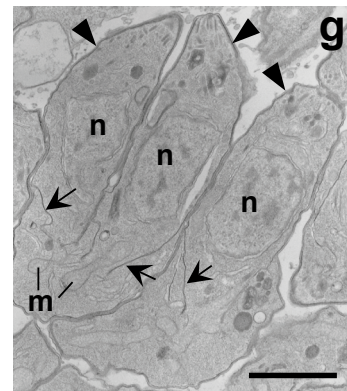

Supplement: Figure S12 — Ultrastructural observations of TgNCR1-deficient parasites. Transmission EM of TgNCR1-deficient parasites infecting HFF for 24 h. A. Replicating parasites were frequently observed for the ΔNCR1 strain, indicative of synchronous division. Images from panel a to g illustrate progressive views of parasite endodyogeny, presenting similarities with wild-type parasites. Arrows pinpoint IMC scaffolds. a, apicoplast; Go, Golgi; m, mitochondrion; mi, micronemes; n, nucleus; r, rhoptries. Bars are 1 µm. (PDF) [file ppat.1002410.s012.pdf]

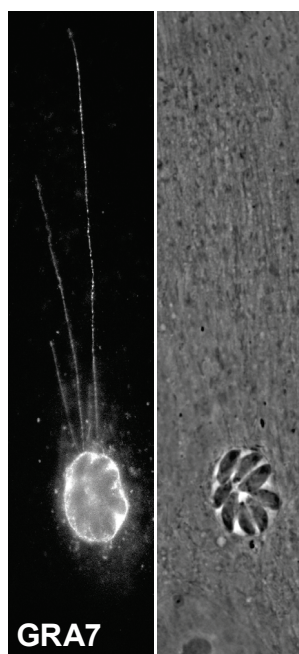

Supplement: Figure S13 — Demonstration of tubular extensions from the PV of wild-type parasites (RH strain). Immunofluorescence assays using anti-GRA7 antibodies on Toxoplasma-infected cells after 24 h. Note the presence of at least three PV membrane extensions into the host cytosol. (PDF) [file ppat.1002410.s013.pdf]
