# Supplementary material for: Deficiency of a Niemann-Pick, Type C1-related Protein in Toxoplasma Is Associated with Multiple Lipidoses and Increased Pathogenicity
Source: PLoS Pathog. 2011 Dec 8;7(12):e1002410. doi: 10.1371/journal.ppat.1002410 (PMC3234224; doi:10.1371/journal.ppat.1002410)
Supplement: Table S1 — Analysis of phospholipid composition in the ΔNCR1 and parental strains by mass spectrometry. Quantitative analysis of MRM spectra from the ΔNCR1 and parental strains showing levels of various species of phospholipids. Statistically significant differences in the amounts of few species of phosphatidylethanolamine and phosphatidylserine were detected in TgNCR1-deficient parasites. Data are cps means ± SD from 3 independent populations from each strain. (PDF) [file ppat.1002410.s014.pdf]

	Parental		ΔNCR1		
**Phosphatidylcholine (PC)**					
d18:0/16:0	3.10E+07	1.25E+06	3.13E+07	1.29E+07	
d18:0/18:0	6.08E+05	5.93E+04	6.68E+05	2.94E+05	
d18:0/20:0	1.57E+05	7.66E+04	2.09E+05	1.40E+05	
d18:0/16:1	5.31E+06	5.20E+05	4.42E+06	2.40E+06	
d18:0/18:1	3.08E+07	3.17E+06	2.79E+07	1.15E+07	
d18:0/18:2	7.08E+06	1.23E+06	6.90E+06	3.50E+06	
d18:0/20:4	1.71E+07	5.53E+06	1.75E+07	7.91E+06	
**Phosphatidylethanolamine (PE)**	Average	S.D.	Average	S. D.	
d18:0/16:0	1.20E+06	5.89E+05	1.32E+06	6.69E+05	
**d18:0/18:0**	**1.74E+04**	**1.20E+04**	**5.14E+04**	**7.60E+03**	**(P<0.049)**
**d18:0/20:0**	**2.15E+04**	**4.11E+03**	**7.14E+04**	**1.68E+04**	**(P<0.021)**
d18:0/16:1	1.19E+07	4.42E+06	1.36E+07	7.41E+06	
d18:0/18:1	2.75E+06	1.35E+06	4.87E+06	2.14E+06	
d18:0/18:2	1.24E+07	4.03E+06	1.65E+07	6.75E+06	
d18:0/20:4	7.94E+05	4.18E+05	1.09E+06	6.49E+05	
**Phosphatidylserine (PS)**					
d18:0/16:0	1.67E+04	6.82E+03	1.97E+04	4.18E+03	
**d18:0/18:0**	**9.32E+03**	**7.60E+03**	**2.48E+04**	**6.07E+03**	**(P<0.033)**
d18:0/16:1	2.61E+05	1.07E+05	2.80E+05	6.13E+04	
d18:0/18:1	1.95E+04	8.73E+03	1.74E+04	6.65E+03	
d18:0/18:2	2.70E+05	1.21E+05	2.75E+05	7.74E+04	
d18:0/20:4	3.11E+04	8.74E+03	5.16E+04	1.08E+04	
**Phosphatidylinositol (PI)**					
d18:0/16:1	2.84E+04	1.25E+04	3.09E+04	3.30E+04	
d18:0/18:1	3.09E+04	1.22E+04	2.70E+04	2.60E+04	
d18:0/18:2	9.42E+03	5.95E+03	9.98E+03	8.64E+03	
d18:0/20:4	1.41E+04	3.01E+03	1.64E+04	1.23E+04	
