# Supplementary material for: Deficiency of a Niemann-Pick, Type C1-related Protein in Toxoplasma Is Associated with Multiple Lipidoses and Increased Pathogenicity
Source: PLoS Pathog. 2011 Dec 8;7(12):e1002410. doi: 10.1371/journal.ppat.1002410 (PMC3234224; doi:10.1371/journal.ppat.1002410)
Supplement: Table S2 — Primers used in this study. (PDF) [file ppat.1002410.s015.pdf]

Name	Sequence 5' to 3' (restriction sites in bold)
F-TgNCR1-P1	ACT**GAATTC**GAAATGGAGAAGAATTGTAACAGCGTGGCAGGCC
R-TgNCR1-P2	CAT**AAGCTT**GACCCCCACTCCATGTTCTTCCTTCCCTG
F-hNPC1$_{1214\text{-}1278}$	CCACCCTGTTGTCGATTCTCGCACTTTTTGCCAAATCTCAAATTTTCCAG ATATTCTACTTCAG
R-hNPC1$_{1214\text{-}1278}$	CAT**AAGCTT**TTATCACTAGAAATTTAGAAGCCGTTCGCGCTCTG
F-*myc*-TgNCR1$_{1\text{-}1101}$	ACT**GAATTC**GAAATGGAACAAAAACTCATCTCAGAAGAGGATCTGGAG AAGAATTGTAACAGCGTGGCAGGCC
R-TgNCR1$_{1\text{-}1101}$ (R-*myc*-TgNCR1$_{938\text{-}1100}$)	CTGAAGTAGAATATCTGGAAAATTTGAGATTTGGCAAAAAGTGCGAGA ATCGACAACAGGGTGG
F-*myc*-TgNCR1$_{938\text{-}1100}$	ACT**GAATTC**GAAATGGAACAAAAACTCATCTCAGAAGAGGATCTGATG CCGCACAGGGACAATACCACAAC
F-HA-TgNCR1	ACT**GGATCC**GACAAAATGGAGAAGAATTGTAACAGCTACCCATACGAC GTCCCAGACTACGCTGTGGCAGGCCACGCCGGAACTCTTATCAAG
R-YFP-TgNCR1	TCA**CCTAGG**TTATCACTAGACCCCCACTCCATGTTCTTCCTTCCCTG
F-TgNCR1$_{D571N}$	CCTGGCCGGACTCAGCTTGAACGCTCGACGCGAAGCCCG
R-TgNCR1$_{D571N}$	CGGGCTTCGCGTCGAGCGTTCAAGCTGAGTCCGGCCAGG
F-TgNCR1$_{P913A}$	GAAAACGTGGCTGGAAGGAGACGCCATCGGACAAAACTTTAGCACC
R-TgNCR1$_{P913A}$	GGTGCTAAAGTTTTGTCCGATGGCGTCTCCTTCCAGCCACGTTTTC
F-TgNCR1$_{I957T}$	TACTGGCTAAAGGAAGGAAAAGATACTGTCAGCGCCGGGAAGCCG
R-TgNCR1$_{I957T}$	CGGCTTCCCGGCGCTGACAGTATCTTTTCCTTCCTTTAGCCAGTA
F-TgNCR1$_{L1100V}$	CCCTGTTGTCGATTCTCGCAGTTGCGGGGTCACCGAAATACATTC
R-TgNCR1$_{L1100V}$	GAATGTATTTCGGTGACCCCGCAACTGCGAGAATCGACAACAGGG
KOPCR-1	CAGCGAGTGTTCGCGGGCGCTACG
KOPCR-2	GACGGTGTGTCAGTGGTGGAATTCACGTAC

KOPCR-3	GCAACTCGGACTGGCGTGCTTGCTCACCGACCACAGCACGAAACCTTGC ATTCAAACCCGCCCGCGGAAG
KOPCR-4	CTTCCGCGGGCGGGTTTGAATGCAAGGTTTCGTGCTGTGGTCGGTGAGC AAGCACGCCAGTCCGAGTTGC
KOPCR-5	GTTCTGGCAGGCTACAGTGACACCGCGATTATCTGTCCTTTAGCAATGA CGCACAGTGTCAGTTGTC
KOPCR-6	GACAACTGACACTGTGCGTCATTGCTAAAGGACAGATAATCGCGGTGTC ACTGTAGCCTGCCAGAAC
P1	GGCTAACACAATTTTCACCGGCGTTTAGTTTCAGAAGA
P2	GACTGCGAACAGCAGCAAGATCGGATC
P3	AGCACGAAACCTTGCATTCAAACCCGCCC
P4	CGCGGTGTCACTGTAGCCTGCCAG
P5	GTATGATCATCCCTGGCTTTTTATCATGGTGTCTTTGC
P6	CGCGTCGAGCGTTCAAGCTGAG
F-haNTP	ACTACTAGTATGGAGAAGAATTGTAACAGCGTGGCAGGCC
R-haNTP	TCATTAATTAATCAGACCCCCACTCCATGTTCTTCCTTCCCTG
F-NCRHA	ACT**GGATCC**GACAAAATGGAGAAGAATTGTAACAGCGTGGCAGGCC
R-NCRHA	TCA**CCTAGG**TTATCACTAAGCGTAGTCTGGGACGTCGTATGGGTAGAC CCCCACTCCATGTTCTTCCTTCCCTG
F-NPC-sb-1	CACCCTCACCGAGTTCGGTGAGCG
R-NPC-sb-1	CGCGGTGTCACTGTAGCCTGCCAG
Ncomp-3	CTGGCGTGCTTGCTCACCGACCACATGGAGAAGAATTGTAACAGCGTG GCAGGCC
Ncomp-4	GGCCTGCCACGCTGTTACAATTCTTCTCCATGTGGTCGGTGAGCAAGCA CGCCAG
Ncomp-5	GGAAGGAAGAACATGGAGTGGGGGTCTGAATTATCTGTCCTTTAGCAA TGACGCACAGTGTCAGTTG
Ncomp-6	CAACTGACACTGTGCGTCATTGCTAAAGGACAGATAATTCAGACCCCCA

	CTCCATGTTCTTCCTTCC
P7	GTCCGCGACAACCGGGACAATTTCGTTC
P8	ACT**GGATCC**ATGGTGTCTTTGCTTGCTACGGCGGGCATG
P9	CAT**AAGCTT**TTAATCCAATGTTGACACGCGCTTCAGTTCATCG
